# How DNA Barcodes Complement Taxonomy and Explore Species Diversity: The Case Study of a Poorly Understood Marine Fauna

**DOI:** 10.1371/journal.pone.0021326

**Published:** 2011-06-16

**Authors:** Jun Chen, Qi Li, Lingfeng Kong, Hong Yu

**Affiliations:** Fisheries College, Ocean University of China, Qingdao, China; Centre National de la Recherche Scientifique, France

## Abstract

**Background:**

The species boundaries of some venerids are difficult to define based solely on morphological features due to their indistinct intra- and interspecific phenotypic variability. An unprecedented biodiversity crisis caused by human activities has emerged. Thus, to access the biological diversity and further the conservation of this taxonomically muddling bivalve group, a fast and simple approach that can efficiently examine species boundaries and highlight areas of unrecognized diversity is urgently needed. DNA barcoding has proved its effectiveness in high-volume species identification and discovery. In the present study, Chinese fauna was chosen to examine whether this molecular biomarker is sensitive enough for species delimitation, and how it complements taxonomy and explores species diversity.

**Methodology/Principal Findings:**

A total of 315 specimens from around 60 venerid species were included, qualifying the present study as the first major analysis of DNA barcoding for marine bivalves. Nearly all individuals identified to species level based on morphological traits possessed distinct barcode clusters, except for the specimens of one species pair. Among the 26 individuals that were not assigned binomial names *a priori*, twelve respectively nested within a species genealogy. The remaining individuals formed five monophyletic clusters that potentially represent species new to science or at least unreported in China. Five putative hidden species were also uncovered in traditional morphospecies.

**Conclusions/Significance:**

The present study shows that DNA barcoding is effective in species delimitation and can aid taxonomists by indicating useful diagnostic morphological traits, informing needful revision, and flagging unseen species. Moreover, the BOLD system, which deposits barcodes, morphological, geographical and other data, has the potential as a convenient taxonomic platform.

## Introduction

The Veneridae (Rafinesque, 1815), known as venus clams, is the most speciose family of heterodont bivalve mollusks [1]. Similar to other heterodont bivalves, larval venerids are planktonic and adults live in substrate environments such as mud, coarse sand, and gravel, and some of them even burrow in weathered rock and coral reefs (e.g., genus *Irus* Schmidt, 1818). To adapt to various substrate environments, their burrowing behavior has led to extensive parallelism of interspecific morphological variability, as well as pronounced intraspecific ecophenotypes. As a result, the intra- and interspecific phenotypic variability of many venerids are indistinct or even overlapping. Therefore, species boundaries of these clams are difficult or even impossible to define accurately based solely on morphological features. Many taxonomic experts disagree regarding the subjective interpretation of variable morphological characters and the validity of species. For some notorious closely related species complexes, even quite varying opinions are held by the same conchologists at their different stages. Thus, Veneridae comprises one of the least understood and most poorly defined molluscan taxa as espoused by Mikkelsen et al. [2].

Considering the species boundaries of some venerids are difficult to delimit based on morphology, additional ecological, reproductive, and other biological data should be employed. However, large-scale investigations using these methods are unlikely launched for this extremely speciose family due to the dwindling of trained taxonomists, as well as costly and time-consuming data collection. An unprecedented biodiversity crisis caused by human activities, such as overharvesting, habitat degradation, global warming, pollution, biological invasions, and other stressors, have emerged in the past decades [3]–[5]. Accurate species delimitation and documentation is vital to accessing biological diversity and furthering conservation. Therefore, a heuristic and high-throughput proxy, that can facilitate the determining of conditions that merit more detailed taxonomic revisions and further aid in focusing the efforts of taxonomists in characterizing biodiversity, should be developed.

The term DNA barcoding was coined by Hebert and colleagues for the use of a standardized DNA region as a tag to fast and reliably identify known species and to aid in the discovery of undescribed species [6], [7]. The 5′ end of the mtDNA cytochrome c oxidase I (COI) gene has been suggested by the Barcode Initiative as a barcode sequence for animal species [8]. To date, this DNA fragment has proved its effectiveness in species identification for the major metazoan animal clades, including both vertebrates (e.g., [9]–[11]) and invertebrates (e.g., [12]–[14]). The efficiency of this barcode marker in the detection of cryptic species has also been well documented in a large array of animal taxa (e.g., [14]–[16]). COI-based barcoding has also revealed very high performance for bivalve groups, albeit only a few species were included [17]–[20]. According to a recent review by Zink and Barrowclough [21], the signal from mtDNA is rarely contradicted by supplemental analyses at nuclear markers. Various recent research have also indicated that divergent barcode clusters indeed correspond to reproductively isolated groups, proving a link between DNA barcode and the biological species [22]–[25]. Hence, this promising standardized molecular approach may have the power to play the role in broadly examining species boundaries of venerids.

**Figure 1:**
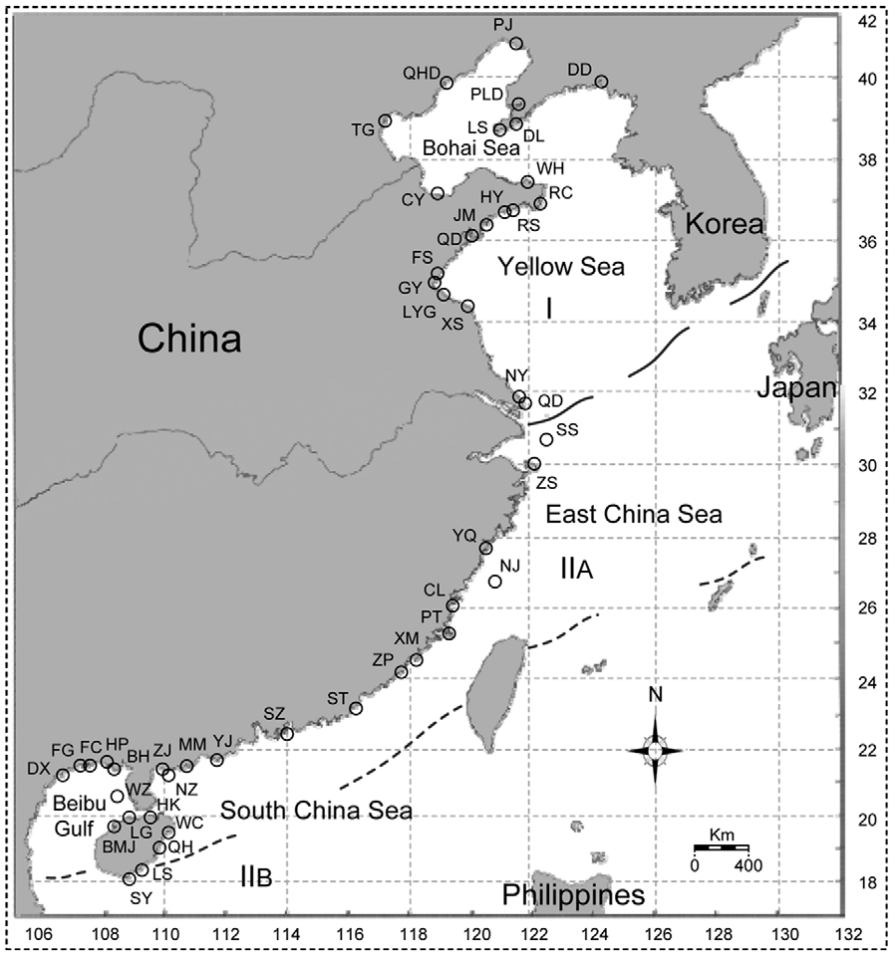
Distribution of locations for the 315 specimens sampled along the coast of mainland China. See Table S1 for the detailed sampling information. Demarcation of marine molluscan faunal regions of China is mapped. I: Far East Subregion of North Pacific Region, IIA: Sino-Japanese Subregion of Indo-West Pacific Region, and IIB: Indo-Malayan Subregion of Indo-West Pacific Region.

In the present study, Chinese venerid fauna was chosen to examine whether DNA barcoding is sensitive enough to reveal discrete biological entities, and how this molecular biomarker complements taxonomy and explores species diversity. As one of the most extensive coastline in the Western Pacific Region, the coastline of China has extensive latitudinal range, well-characterized oceanography, and dramatic geological history [26]. Two molluscan faunal regions have been demarcated along the coast of China (Figure 1), with approximate 100 venerid species reported [27], [28]. Among these venerid species, only eight were described in the last century, and just two were described by local taxonomic experts (i.e., Chinese malacologists) [28]. This situation suggests that the traditional taxonomy of Chinese venerid fauna is substantially outdated and that venerid biodiversity might be severely underestimated. Therefore, genetic barcoding analysis presented herein also provides an ideal opportunity to offer fresh insights into the taxonomy and biodiversity of this poorly understood fauna.

## Materials and Methods

### Biological Material Sampling

The samples included in the present study were collected along the coast of China from April 2004 to May 2010 (Figure 1 and Table S1). These samples were stored in 95% ethanol and deposited as voucher specimens in Fisheries College, Ocean University of China. Species were delimited *a priori* based on currently published taxonomic literature. In cases when the identification was difficult, some taxonomic specialists were consulted. However, some specimens still could not be reliably assigned binomial names. These specimens were just identified to taxonomic level as low as possible (Table S1). The classification scheme proposed by Habe [29] was followed throughout this study. Photographs of specimens used in this study as well as collecting data are available in the project ‘Bivalves along the Coast of China’ on the Barcode of Life Data System (BOLD) at http://www.barcodinglife.org/.

### Molecular Data Collection

A double mechanism of transmission, called “doubly uniparental inheritance” (DUI), that both male (M-type) and female (F-type) mtDNA are inherited uniparentally, is known in some bivalves including venerid *Ruditapes philippinarum* [30]. Infrequent M-type mtDNA is restricted to male gonadal tissue [31]. Thus, to avoid collecting M-type mtDNA, total genomic DNA was extracted only from the adductor muscle tissue using a modified phenol-chloroform procedure described by Li et al. [32].

**Figure 2:**
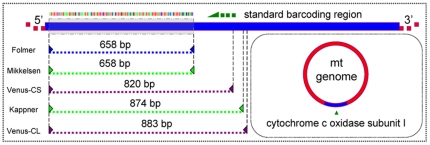
A diagrammatic representation of the standard barcoding region and the primer binding sites used in this study.

Polymerase chain reaction (PCR) was performed in a total volume of 50 µl with 2 U Taq DNA polymerase, 100 ng template DNA, 1 µM each primer, 200 µM of each dNTP, 1× PCR buffer, 2 mM MgCl_2_, and 4% DMSO. The PCR cycles were carried out under the following conditions: an initial denaturation for 3 min at 94°C, followed by 35 cycles of 40 s at 94°C, 40 s at primer-specific annealing temperatures (Table 1), 40 s at 72°C, and with a final 5-min extension at 72°C. Kappner's [33] and Mikkelsen's [2] primers were employed to amplify COI when Folmer's [34] primers failed. However, some taxa still could not be sequenced successfully. Thus, two sets of customized primer cocktails for venerids were developed to better match this mitochondrial gene region (Figure 2 and Table 1). Sequencing was performed in both directions, and the complementary DNA sequence strands were edited, assembled, and merged into consensus sequences using the software program Seqman II 5.07 (Lasergene, DNASTAR). All sequences were deposited in GenBank under accession numbers from HQ703031 to HQ703342 and BOLD with numbers from BCC001-10 to BCC315-10 (See Table S1 for details).

**Table 1:** Primer sequences and annealing temperatures used to amplify DNA barcodes in this study.

Set name	Primer name	Sequence (5′–3′)	Annealing T (°C)	Source
Folmer			48–52	
	LCO1490†	GGTCAACAAATCATAAAGATATTGG		[53]
	HCO2198<$>\raster="rg1"<$>	TAAACTTCAGGGTGACCAAAAAATCA		[53]
Mikkelsen			48	
	COIF-ALT†	ACAAATCAYAARGAYATYGG		[6]
	COIR-ALT<$>\raster="rg1"<$>	TTCAGGRTGNCCRAARAAYCA		[6]
Venus-CS			48–50	
	COIF-ALT†	ACAAATCAYAARGAYATYGG		[6]
	COIF-VSA†	ACCAATCATAAAGATATTGG		Modified from [52]
	COIRVBSI<$>\raster="rg1"<$>	CCNAYHGTAAAYATATGRTG		This study
	COIRVBSO<$>\raster="rg1"<$>	CCDRCNGTAAAYATRTGATG		This study
Kappner			48–54	
	LCO1490-Ven†	ATTATTCAGAACCAATCATAAAGATATTGG		[52]
	HCOI900-Ven<$>\raster="rg1"<$>	TGTAGGAATAGCAATAATAAAAGTTAC		[52]
Venus-CL			48–50	
	COIF-ALT†	ACAAATCAYAARGAYATYGG		[6]
	COIF-VSA†	ACCAATCATAAAGATATTGG		Modified from [52]
	COIRVBLP<$>\raster="rg1"<$>	CCTGTAGGAATAGCAATAAT		This study
	COIRVBLJ<$>\raster="rg1"<$>	CCWGTWGGRACAGCAATAAT		This study

† Forward primer.

^<$>\raster="rg1"<$>^Reverse primer.

As complementary data, additional barcodes were obtained from BOLD. All 320 public records (Document S1) were downloaded, and 310 sequences longer than 400 bp in length were merged with the sequences collected by ourselves (hereafter referred to as the “original dataset”), creating a complete dataset of 622 sequences (hereafter referred to as the “complete dataset”).

### DNA Barcoding Analyses

Genetic distances were calculated using the Kimura 2-parameter (K2P) model [35] in MEGA 4.1 [36]. The analyses were performed as follows: (a) Maximum intraspecific distances and minimum interspecific distances of individuals, which were reliably assigned binomial names *a priori*, were calculated; (b) Distances were calculated at the species, genus, subfamily, and family level respectively based on the original dataset; and (c) Pairwise distances, based on the original dataset and the complete dataset respectively, were calculated among venerids regardless of their binomial names.

For the threshold-based approach, the sequences were also grouped into provisional clusters as molecular operational taxonomic units (MOTUs) using the alignment-based parametric software TaxonDNA 1.6.5 [37]. The MOTUs were defined for both the original and the complete datasets. The analyses were implemented at cut-off values ranging from 0–25% sequence divergence.

A neighbor-joining (NJ) tree was reconstructed for the complete dataset using the K2P model with a bootstrap support analysis (1000 replicates) in MEGA. To infer the systematic relationships among species of certain groups more effectively, the maximum likelihood (ML) approach was applied using PhyML 3.0 with 1000 bootstrap replicates [38]. The best-fit models of nucleotide substitution were inferred using jModeltest 0.1.1 [39].

## Results

A total of 315 venerid specimens were analyzed, among which, 289 were defined into 51 species based on morphological characters. Appropriate binomial names were not assigned to the remaining 26 individuals because they were either still undescribed or were unable to be reliably identified based on available reference materials. These specimens were provisionally assigned into 13 morphologically distinct groups.

**Figure 3:**
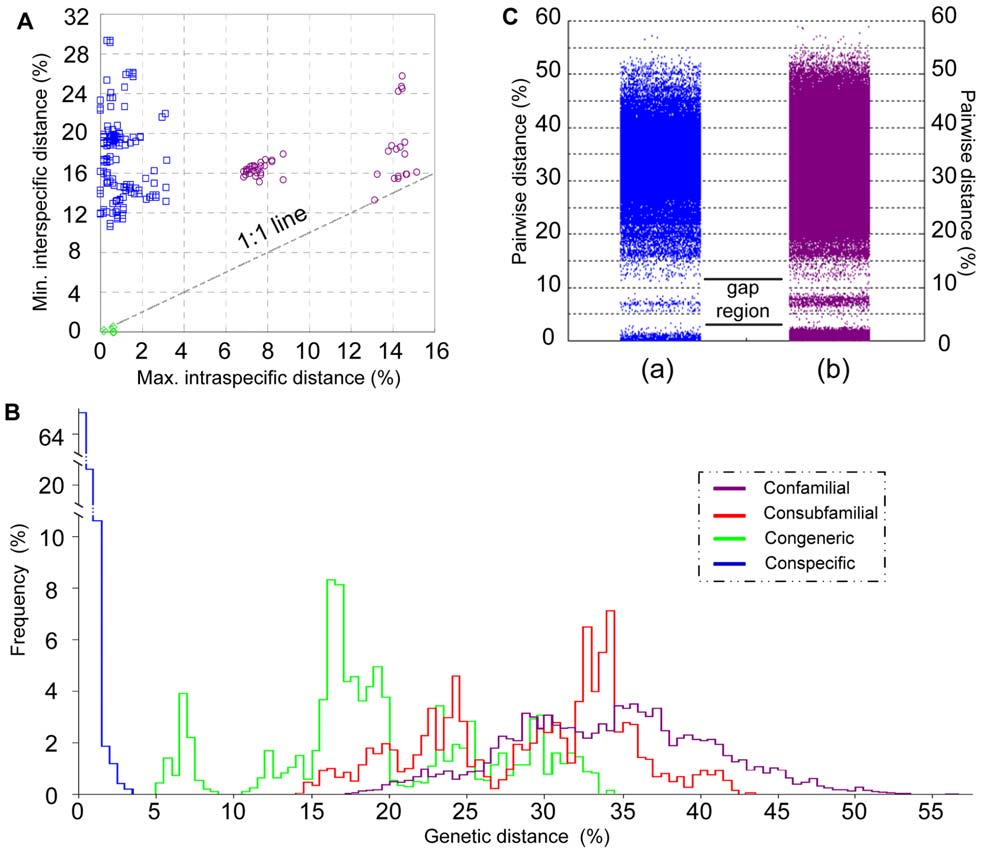
Statistical results of genetic distance analyses. A. Maximum intraspecific distances versus minimum interspecific distances. Performances were based on individuals reliably identified *a priori* to the species level. □: Species were monophyly with low genetic diversity. ○: Species divided into two well-separated clusters. ◊: Individuals of species pairs exhibited very low level of genetic diversity. B. Distribution of distances among conspecific, congeneric, consubfamilial, and consubfamilial individuals. Species assignments were the final assignments according to our barcoding analysis. C. Pairwise distances within Veneridae. (a): 48516 pairs from the original dataset. (b): 193131 pairs from the complete dataset.

Majority of the individuals that were reliably identified to species level based on their morphological characters displayed very low ratios of maximum intraspecific distance to minimum interspecific distance (Figure 3a). However, a high level of intraspecific variation was observed in five morphospecies (*Gafrarium dispar*, *Circe scripta*, *Meretrix petechialis, Paphia gallus* and *Periglypta puerpera*) (Table 2), and individuals of *Phacosoma biscoticum* and *P. fibulum* showed evidence of extremely low level of interspecific genetic diversity. These individuals, that displayed uncommon maximum intraspecific to minimum interspecific distance ratios, might be suffering wrong species delimitations. Thus, some specimens were reallocated to new provisional species groups. Based on the final species delimitations, the genetic distances among individuals that shared the same assignments ranged from 0 to 3.17%, and the adjusted congeneric comparisons were between 5.45% and 34.17% (Figure 3b). The mean congeneric distance (19.64%) was approximately 40-fold higher than the conspecific variation (0.49%). Nearly all congeneric distances were higher than 10%, except for the species pairs *Macridiscus aequilatera* and *M. semicancellata, M. petechialis* A and B, and *C. scripta* A and B. Although no large gap between intra- and interspecific genetic divergence variation was observed, the K2P distances ranging from 4% to 10% were regarded as the barcoding “gap region” because very few barcode distances fell in this rank. The 310 sequences downloaded from the BOLD database were subsequently included into our pairwise genetic distance analyses, with this broader sampling not evidently filling this barcoding gap region (Figure 3c).

**Table 2:** Barcode divergence statistics for the five apparent cases of cryptic variation (%).

MOTU number	Defined a *priori*	Within MOTU			Between MOTU			Final assignment
		Min.	Mean	Max.	Min.	Mean	Max.	
16	*Gafrarium dispar*	0.00	0.95	2.04	13.26	14.32	15.14	*Gafrarium dispar* A
19		—	—	—				*Gafrarium dispar* B
7	*Circe scripta*	0.15	0.72	1.40	7.70	8.11	8.75	*Circe scripta* A
8		0.00	0.00	0.00				*Circe scripta* B
28	*Meretrix petechialis*	0.15	0.52	1.15	6.42	6.96	7.67	*Meretrix petechialis* A
72		0.00	0.79	1.70				*Meretrix petechialis* B
116	*Paphia gallus*	—	—	—	14.42	14.36	14.43	*Paphia gallus* A
96		0.15	0.20	0.31				*Paphia gallus* B
102	*Periglypta puerpera*	0.00	0.50	1.17	13.37	14.15	14.58	*Periglypta puerpera* A
82		—	—	—				*Periglypta puerpera* B

**Figure 4:**
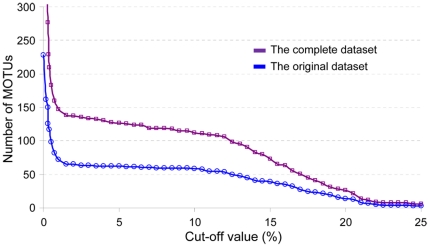
Variation in the numbers of MOTUs defined at cut-offs from 0 to 25% for barcodes.

Variations in the richness of the MOTUs delimited at cut-offs ranging from 0 to 25% for venerid barcodes are shown for both the original dataset and the complete dataset in Figure 4. Both of these two datasets exhibited a plateau in MOTU numbers consistent with the barcoding gap region. The MOTUs designated by TaxonDNA should have the highest correct assignment rate at the gap region. Considering that a lower cut-off value can more efficiently screen potential lumping taxa and uncover hidden biological diversity, our final MOTU name assignments were adopted at the restrictive arbitrary value of 4%. At this cut-off threshold, 62 and 131 MOTUs were recognized for the original dataset and the complete dataset, respectively.

**Figure 5:**
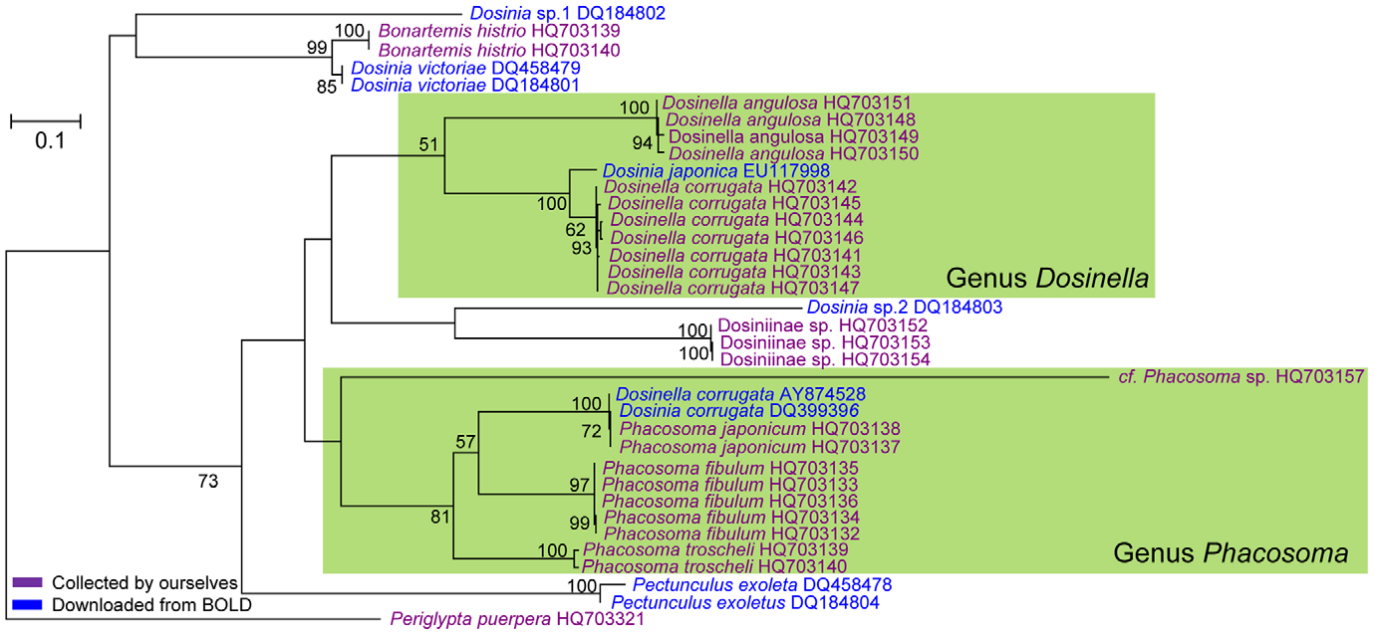
A maximum likelihood tree of barcodes from individuals of the subfamily Dosiniinae. Numbers near the nodes indicate ML bootstrap support. Support values less than 50 are not shown. Species names and GenBank accession numbers are given at branch tips. *Periglypta puerpera* is selected as outgroup.

**Figure 6:**
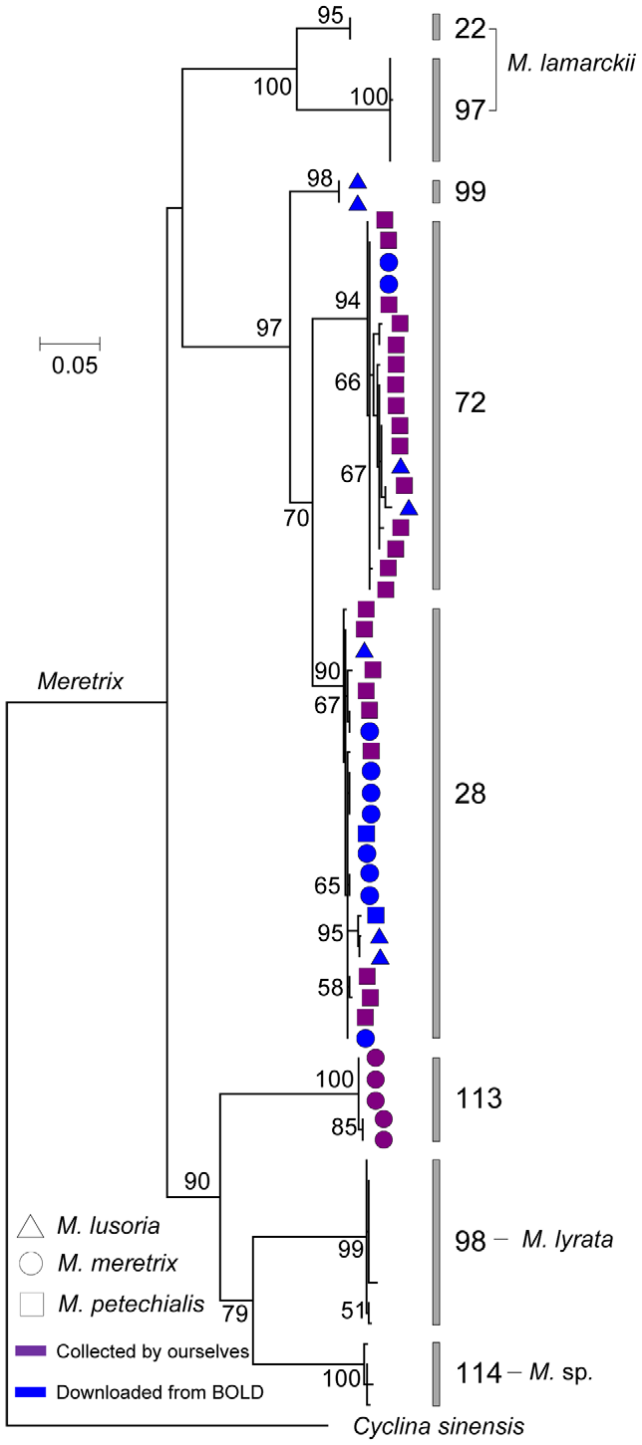
A maximum likelihood tree of barcodes from individuals of the genus *Meretrix*. Numbers near the nodes indicate ML bootstrap support. Support values less than 50 are not shown. The MOTU numbers of each barcode clusters are given at branch tips. *Cyclina sinensis* is selected as outgroup.

The five morphospecies with high conspecific variations respectively formed two monophyletic clusters in our NJ analysis (Figure S1). When the barcodes downloaded from BOLD database were considered, four additional cases (*Globivenus toreuma*, *M. lamarckii*, *Protothaca jedoensis*, and *Pitarina japonica*) that might represent cryptic species were revealed. Moreover, amounts of barcodes from BOLD did not nest among their putative conspecifics but fell far afield within the clusters formed by other species (Figure S1; see Figure 5 and Figure 6 for two obvious cases).

## Discussion

### Utility of COI for Species Delimitation

Our analyses indicate that the individuals, that were reliably assigned binomial names *a priori*, possessed distinct COI sequences, except for the five individuals of *P. biscoticum* and *P. fibulum*. Among the individuals which were unable to assign binomial names, 11 formed five monophyletic clusters. These units of diversity are likely species new to science or at least unreported in China because of their significant morphological distinctness to well-established species from the coast of China. The remaining 12 specimens cohesively nested unambiguously within a species genealogy respectively. Considering that morphologically ambiguous and/or taxonomically controversial species were readily detected and potential cryptic diversities were also efficiently uncovered by barcodes herein, this genetic maker is sensitive for the delimitation of venerid species.

One of the earlier criticisms on DNA barcoding was that specimen sampling is not sufficiently exhaustive [40]. Herein, when data downloaded from BOLD were included our analyses, the barcoding region still existed (Figures 3c and 4). Heteroplasmy (e.g., DUI) is another reason for deep mtDNA divergence within the same biological species, even the same individuals. Our results show that M-type and F-type barcodes of *R. philippinarum* formed separate clusters (Figure S1). Considering the barcode array for one species remains distinct from the others, geographical differentiation and heteroplasmy apparently does not pose a significant issue for tree-based barcoding analysis.

### The Role of Local-scale DNA Barcoding Projects

Although animal groups including numerous marine bivalves whose morphological taxonomy are in chaos would greatly benefit from DNA barcoding, no global barcoding campaign for bivalves have been performed as in other relatively well-understood animal groups. Nevertheless, building databases for regional fauna and feedback between DNA and traditional taxonomy will also assist us in refining the current taxonomic status and understanding biodiversity.

#### 1. Mapping Diagnostic Morphological Traits

**Figure 7:**
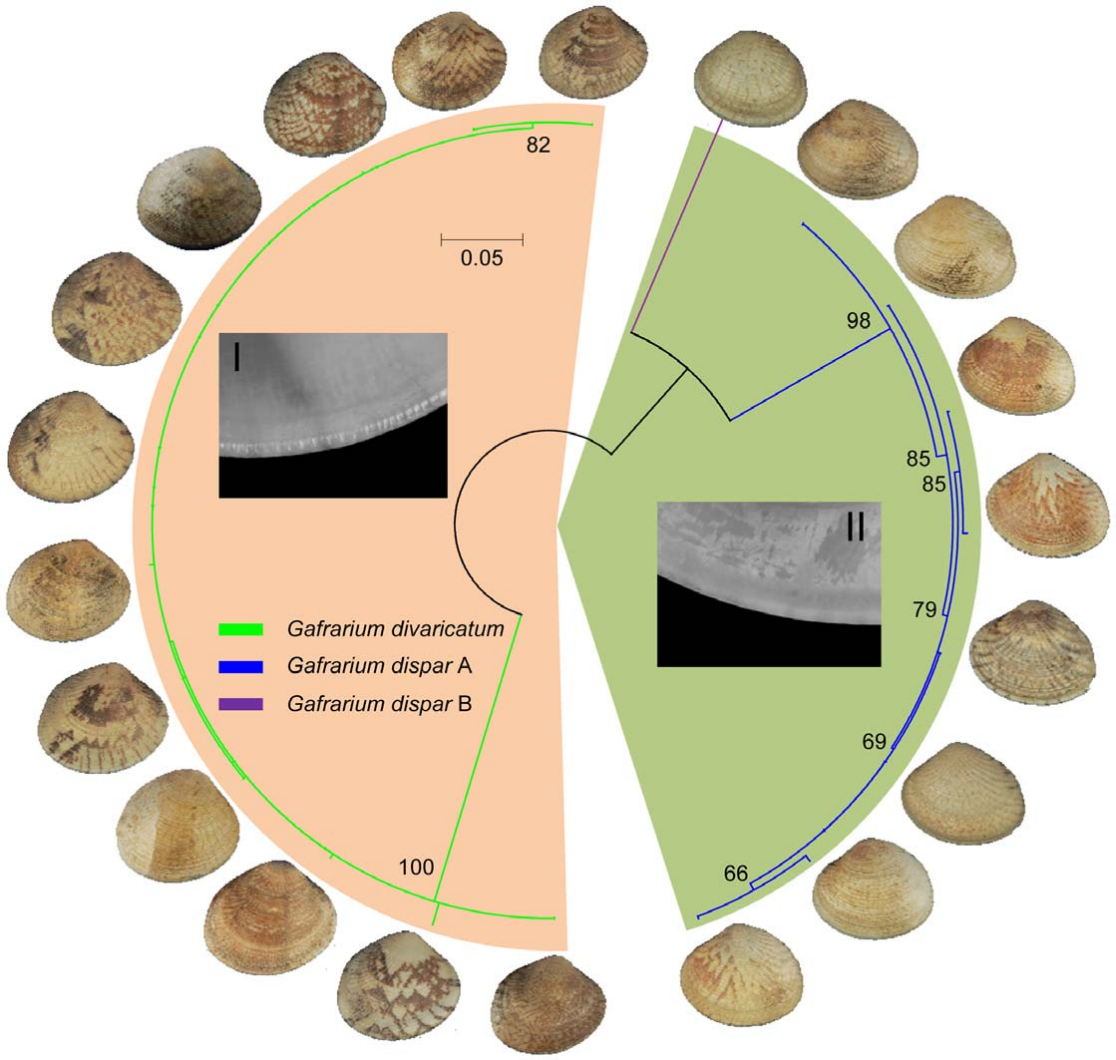
A maximum likelihood tree of barcodes from two conchologically similar *Gafrarium* morphospecies. Numbers near the nodes indicate ML bootstrap support. Support values less than 50 are not shown. Distinguishing morphological traits were mapped into barcode clusters, I: crenulated interior shell margin of *G. divaricatum*, and II: smooth interior shell margin of *G. dispar* A and B.

Intermediate types exist in numerous venus siblings, even in some taxon assemblages without controversies at the species level. Some intermediate types are too misleading to correctly determine their binomial names even for taxonomic specialists because the key characters for separating sister species are difficult to determine [28]. Mapping morphological characters into genetic clusters is an additional way to assist taxonomists in determining diagnostic traits. The morphology of some individuals of *G. dispar* and *G. divaricatum*, for example, are intermediate. Based on their well-separated barcode clusters, all *G. divaricatum* specimens were found to have crenulations in their interior shell margin in contrast to individuals of *G. dispar* specimens (Figure 7). This conchological trait was ignored in most former taxonomic literature. After re-examining more than 400 specimens deposited in our laboratory, we found this character was unambiguous for separating these two species.

#### 2. Informing Revision of Current Taxonomy

Given that many taxonomic experts disagree regarding the interpretation of variable morphologic characters to keep species apart or together in some venerid groups, shell morphology apparently could not provide additional useful information to clarify their biospecies status. The barcoding approach provides new insights in examining species boundaries herein. The number of species that should be recognized within the *C. scripta* complex is controversial, whether one species [41], [42], two species [29], or three species [28]. Two barcode clusters were recovered here but they do not represent any morphospecies of traditional taxonomy. Ongoing work is investigating this issue using both molecular and morphological methods. *Phacosoma biscoticum* and *P. fibulum*, which are two independent species in taxonomic history, lacked intraspecific divergence. To rigorously examine their biospecies status, the sampling should be more adequate and nuclear markers should be added in future studies. *Paphia schnelliana*, described in the Chinese literature [28], [42], [43], possesses relatively distinct morphological characters from *P. schnelliana* (Dunker, 1862), and individuals of *P. schnelliana* Zhuang (2001) clustered with *P. amabilis* in our barcode tree. Therefore, the *P. schnelliana* reported in China might be *P. amabilis* rather than *P. schnelliana*, or these two species should probably be synonymized.

#### 3. Flagging Hidden Diversity

Five putative hidden species were flagged in *M. petechialis, C. scripta, G. dispar, P. gallus* and *P. puerpera*. In addition, six unrecognized species in China were uncovered based on morphology and barcodes, except for *Timoclea* sp. whose barcode data are absent. It is quite intriguing to note that four small specimens (*Meretrix* sp.), which were initially inferred as juvenile *M. lyrata* due to the similarity of their shells to that of *M. lyrata* and concurrent sampling of some *M. lyrata* individuals at the same collecting locality, formed an independent cluster in our NJ tree. *Meretrix* sp. was documented as *M. planisulcata*, a new record in China by Xu and Zhang [28]. However, *M. planisulcata* possesses some conchological characters such as inequilateral shells, narrow posterior end and wide hinge plate [44], [45], which are markedly different from *Meretrix* sp. Therefore, the specimens sampled in China likely represent a new species in the genus *Meretrix*.

### Current Limitations of BOLD and Its Potential Roles

Amounts of publicly available BOLD data did not nest among their putative conspecifics in our NJ tree. Most of these specimens might have been misidentified by submitters. For example, the barcodes of *D. corrugata* downloaded from BOLD nested within *P. japonicum* sampled by ourselves, whereas published barcode of *P. japonicum* nested within our *D. corrugate* cluster. Considering that the genera *Phacosoma* and *Dosinella* occupied different positions in the ML tree (Figure 5), we inferred that these records in BOLD likely resulted from misidentifications. On the other hand, species-name discordance might reflect errors in the taxonomic literature of some countries, which is caused by the scarcity of both reliably identified reference collections and expert taxonomists. For example, the present barcoding analyses evidences that the specific name *M. meretrix* has been used for various species, and *M. lusoria* and *M. petechialis* are intertwined with each other as pointed out by Yamakawa et al. [46] and by Yoosukh and Matsukuma [47] (Figure 6).

Given the unreliability of the current venerid reference barcode library in BOLD, identification of venus clams using the BOLD Identification Systems is not prudent at present. However, our barcoding analyses show that BOLD still has the potential to flag unseen species and reveal cases of errors in the taxonomic literature of some countries. Nevertheless, to enable the possibility of scrutinizing the identity of the specimen from which a sequence was obtained, comprehensive morphological and geographical data, and if necessary, other information such as barcode nature (e.g. M-type and introgression), additional independent genetic markers, and the ecological data of voucher specimens must be submitted along with barcodes.

### Conclusion

DNA barcoding was proposed for dual purposes: species identification and species discovery [6], [7]. Considering that barcoding is more sensitive than morphological analysis in some morphologically confusing animal groups, taxonomy disentanglement should be its third role. However, in spite of their potential for efficiently examining species boundaries, barcode clusters fulfill the phylogenetic species concept and are not destined to be biological species [48]. Therefore, when the overlumpings and oversplittings of traditional taxonomy are flagged by barcoding analyses, additional taxonomic methods are required to draw solid conclusions.

## Supporting Information

Table S1 **List of specimens with the classification, collection details, and voucher numbers.** Species names defined based on morphological characters and our barcoding analysis are reported respectively. GenBank accession numbers and BOLD specimen numbers are given in the last tow columns.
(PDF)

Document S1 **FastA file with all 320 public venerid barcodes in the BOLD database.**
(TXT)

Figure S1 **A neighbour-joining tree of 622 COI sequence from venerid species sampled by ourselves and obtained in BOLD, using K2P distances.** Numbers near the nodes indicate NJ bootstrap support. Species names and GenBank accession numbers are given at branch tips.
(PDF)
