# Supplementary material for: How DNA Barcodes Complement Taxonomy and Explore Species Diversity: The Case Study of a Poorly Understood Marine Fauna
Source: PLoS One. 2011 Jun 16;6(6):e21326. doi: 10.1371/journal.pone.0021326 (PMC3116896; doi:10.1371/journal.pone.0021326)
Supplement: Table S1 — List of specimens with the classification, collection details, and voucher numbers. Species names defined based on morphological characters and our barcoding analysis are reported respectively. GenBank accession numbers and BOLD specimen numbers are given in the last tow columns. (PDF) [file pone.0021326.s001.pdf]

Classification	Voucher number	Collection information<br>Locality	Year	Collector	MOTU[§] number	Species<br>Defined a *priori*	Final assignment	GenBank accession number	BOLD specimen number
Callistinae									
	ZB080301	Beihai, Guangxi (BH)	2008	Qi Li & Xiaodong Zheng	9	*Costacallista erycina*	*Costacallista erycina*	HQ703031	BCC001-10
	ZB080302	Beihai, Guangxi (BH)	2008	Qi Li & Xiaodong Zheng	9	*Costacallista eryina*	*Costacallista erycina*	HQ703032	BCC002-10
	ZB080303	Beihai, Guangxi (BH)	2008	Qi Li & Xiaodong Zheng	9	*Costacallista eryina*	*Costacallista erycina*	HQ703033	BCC003-10
	ZB100401	Beihai, Guangxi (BH)	2010	Lingfeng Kong & Jun Chen	9	*Costacallista eryina*	*Costacallista erycina*	HQ703034	BCC004-10
	ZP100101	Pingtan, Fujian (PT)	2010	Qiaozhen Ke	5	*Callista chinensis*	*Callista chinensis*	HQ703035	BCC005-10
	ZP100102	Pingtan, Fujian (PT)	2010	Qiaozhen Ke	5	*Callista chinensis*	*Callista chinensis*	HQ703036	BCC006-10
	DQ080401	Qingdao, Shandong (QD)	2008	Jingbo Wang & Yanwei Feng	15	*Ezocallista brevisiphonata*	*Ezocallista brevisiphonata* [#]	HQ703037	BCC007-10
	DQ080402	Qingdao, Shandong (QD)	2008	Jingbo Wang & Yanwei Feng	15	*Ezocallista brevisiphonata*	*Ezocallista brevisiphonata* [#]	HQ703038	BCC008-10
	DQ080403	Qingdao, Shandong (QD)	2008	Jingbo Wang & Yanwei Feng	15	*Ezocallista brevisiphonata*	*Ezocallista brevisiphonata* [#]	HQ703039	BCC009-10
	DQ080404	Qingdao, Shandong (QD)	2008	Jingbo Wang & Yanwei Feng	15	*Ezocallista brevisiphonata*	*Ezocallista brevisiphonata* [#]	HQ703040	BCC010-10
	ZP060701	Panjin, Liaoning (PJ)	2006	Shikai Liu	30	*Saxidomus purpurata*	*Saxidomus purpurata*	HQ703041	BCC011-10
	ZP060702	Panjin, Liaoning (PJ)	2006	Shikai Liu	30	*Saxidomus purpurata*	*Saxidomus purpurata*	HQ703042	BCC012-10
	ZP060703	Panjin, Liaoning (PJ)	2006	Shikai Liu	30	*Saxidomus purpurata*	*Saxidomus purpurata*	HQ703043	BCC013-10
	ZP060704	Panjin, Liaoning (PJ)	2006	Shikai Liu	30	*Saxidomus purpurata*	*Saxidomus purpurata*	HQ703044	BCC014-10
	SD100201*	Dalian, Liaoning (DL)	2010	Hongtao Nie	30	*Saxidomus* sp.	*Saxidomus purpurata*	HQ703045	BCC015-10
	SD100202*	Dalian, Liaoning (DL)	2010	Hongtao Nie	30	*Saxidomus* sp.	*Saxidomus purpurata*	HQ703046	BCC016-10

	SD100203*	Dalian, Liaoning (DL)	2010	Hongtao Nie	30	*Saxidomus* sp.	*Saxidomus purpurata*	HQ703047	BCC017-10
Chioninae									
	LW061201*	Wenchang, Hainan (WC)	2006	Rongxiang Gao	1	*Anomalodiscus squamosus*	*Anomalodiscus squamosus*	HQ703048	BCC018-10
	LW061202	Wenchang, Hainan (WC)	2006	Rongxiang Gao	1	*Anomalodiscus squamosus*	*Anomalodiscus squamosus*	HQ703049	BCC019-10
	LL061201	Lingao, Hainan (LG)	2006	Rongxiang Gao	1	*Anomalodiscus squamosus*	*Anomalodiscus squamosus*	HQ703050	BCC020-10
	LS080201	Sanya, Hainan (SY)	2008	Qi Li & Xiaodong Zheng	1	*Anomalodiscus squamosus*	*Anomalodiscus squamosus*	HQ703051	BCC021-10
	QW080201	Wenchang, Hainan (WC)	2008	Qi Li & Xiaodong Zheng	10	*Cryptonema producta*	*Cryptonema producta*	HQ703052	BCC022-10
	QW080202	Wenchang, Hainan (WC)	2008	Qi Li & Xiaodong Zheng	10	*Cryptonema producta*	*Cryptonema producta*	HQ703053	BCC023-10
	QB080401*	Beihai, Guangxi (BH)	2008	Lingfeng Kong & Jun Chen	10	*Cryptonema producta*	*Cryptonema producta*	HQ703054	BCC024-10
	QS080201	Sanya, Hainan (SY)	2008	Qi Li & Xiaodong Zheng	10	*Cryptonema producta*	*Cryptonema producta*	HQ703055	BCC025-10
	QB100401	Beihai, Guangxi (BH)	2010	Lingfeng Kong & Jun Chen	10	*Cryptonema producta*	*Cryptonema producta*	HQ703056	BCC026-10
	SF080401	Fanggang, Guangxi (FG)	2008	Lingfeng Kong & Jun Chen	N/A	*Timoclea* sp.	*Timoclea* sp.[#]	N/A	BCC027-10
	SF080402	Fanggang, Guangxi (FG)	2008	Lingfeng Kong & Jun Chen	N/A	*Timoclea* sp.	*Timoclea* sp.[#]	N/A	BCC028-10
	SF080403	Fanggang, Guangxi (FG)	2008	Lingfeng Kong & Jun Chen	N/A	*Timoclea* sp.	*Timoclea* sp.[#]	N/A	BCC029-10
	YH080401	Hebu, Guangxi (HB)	2008	Lingfeng Kong & Jun Chen	128	*Placamen isabellina*	*Placamen isabellina*	HQ703057	BCC030-10
	YH080402	Hebu, Guangxi (HB)	2008	Lingfeng Kong & Jun Chen	128	*Placamen isabellina*	*Placamen isabellina*	HQ703058	BCC031-10
	YH080403	Hebu, Guangxi (HB)	2008	Lingfeng Kong & Jun Chen	128	*Placamen isabellina*	*Placamen isabellina*	HQ703059	BCC032-10
	YB080401	Beihai, Guangxi (BH)	2008	Lingfeng Kong & Jun Chen	128	*Placamen isabellina*	*Placamen isabellina*	HQ703060	BCC033-10
	YB080402	Beihai, Guangxi (BH)	2008	Lingfeng Kong & Jun Chen	128	*Placamen isabellina*	*Placamen isabellina*	HQ703061	BCC034-10
	SB080411	Beihai, Guangxi (BH)	2008	Lingfeng Kong & Jun Chen	106	*Placamen* sp.	*Placamen calophylla*	HQ703062	BCC035-10

	SS080401	Sanya, Hainan (SY)	2006	Rongxiang Gao	106	*Placamen* sp.	*Placamen calophylla*	HQ703063	BCC036-10
	MF080301	Fangcheng, Guangxi (FC)	2008	Lingfeng Kong & Jun Chen	106	*Placamen calophylla*	*Placamen calophylla*	HQ703064	BCC037-10
	JH060701	Haiyang, Shandong (HY)	2006	Qi Li	129	*Protothaca jedoensis*	*Protothaca jedoensis*	HQ703065	BCC038-10
	JG080901	Ganyu, Jiangsu (GY)	2008	Hongtao Nie	129	*Protothaca jedoensis*	*Protothaca jedoensis*	HQ703066	BCC039-10
	JG080902	Ganyu, Jiangsu (GY)	2008	Hongtao Nie	129	*Protothaca jedoensis*	*Protothaca jedoensis*	HQ703067	BCC040-10
	JQ080501	Qingdao, Shandong (QD)	2008	Jun Chen & Jingbo Wang	129	*Protothaca jedoensis*	*Protothaca jedoensis*	HQ703068	BCC041-10
	JQ080502*	Qingdao, Shandong (QD)	2008	Jun Chen & Jingbo Wang	129	*Protothaca jedoensis*	*Protothaca jedoensis*	HQ703069	BCC042-10
	JR100101	Rushan, Shangdong (RS)	2010	Qi Li	129	*Protothaca jedoensis*	*Protothaca jedoensis*	HQ703070	BCC043-10
	JL081201	Lianyugang, Jiangsu (LYG)	2008	Hongtao Nie	26	*Mercenaria mercenaria*	*Mercenaria mercenaria*	HQ703071	BCC044-10
	JL081202	Lianyugang, Jiangsu (LYG)	2008	Hongtao Nie	26	*Mercenaria mercenaria*	*Mercenaria mercenaria*	HQ703072	BCC045-10
	JL081203	Lianyugang, Jiangsu (LYG)	2008	Hongtao Nie	26	*Mercenaria mercenaria*	*Mercenaria mercenaria*	HQ703073	BCC046-10
	JL081204	Lianyugang, Jiangsu (LYG)	2008	Hongtao Nie	26	*Mercenaria mercenaria*	*Mercenaria mercenaria*	HQ703074	BCC047-10
	JL081205	Lianyugang, Jiangsu (LYG)	2008	Hongtao Nie	26	*Mercenaria mercenaria*	*Mercenaria mercenaria*	HQ703075	BCC048-10
	JL081206*	Lianyugang, Jiangsu (LYG)	2008	Hongtao Nie	26	*Mercenaria mercenaria*	*Mercenaria mercenaria*	HQ703076	BCC049-10
Circinae									
	JW080201	Wenchang, Hainan (WC)	2008	Qi Li & Xiaodong Zheng	18	*Gafrarium pectinatum*	*Gafrarium pectinatum*	HQ703077	BCC050-10
	JW080202	Wenchang, Hainan (WC)	2008	Qi Li & Xiaodong Zheng	18	*Gafrarium pectinatum*	*Gafrarium pectinatum*	HQ703078	BCC051-10
	JQ100401	Sanya, Hainan (SY)	2010	Lingfeng Kong & Jun Chen	18	*Gafrarium pectinatum*	*Gafrarium pectinatum*	HQ703079	BCC052-10
	SS100411	Qionghai, Hainan (QH)	2010	Lingfeng Kong & Jun Chen	18	*Gafrarium* sp.1	*Gafrarium pectinatum*	HQ703080	BCC053-10
	TW061101	Wenchang, Hainan (WC)	2006	Rongxiang Gao	20	*Gafrarium tumidum*	*Gafrarium tumidum*	HQ703081	BCC054-10

TW061102	Wenchang, Hainan (WC)	2006	Rongxiang Gao	20	*Gafrarium tumidum*	*Gafrarium tumidum*	HQ703082	BCC055-10
TL080301	Lingao, Hainan (LG)	2008	Qi Li & Xiaodong Zheng	20	*Gafrarium tumidum*	*Gafrarium tumidum*	HQ703083	BCC056-10
SS061211	Sanya, Hainan (SY)	2006	Rongxiang Gao	20	*Gafrarium* sp.2	*Gafrarium tumidum*	HQ703084	BCC057-10
SQ100401	Qionghai, Hainan (QH)	2010	Jun Chen & Jingbo Wang	20	*Gafrarium* sp.2	*Gafrarium tumidum*	HQ703085	BCC058-10
KL080301	Lingao, Hainan (LG)	2008	Qi Li & Xiaodong Zheng	16	*Gafrarium dispar*	*Gafrarium dispar* A	HQ703086	BCC059-10
KL080302	Lingao, Hainan (LG)	2008	Qi Li & Xiaodong Zheng	16	*Gafrarium dispar*	*Gafrarium dispar* A	HQ703087	BCC060-10
KL080303	Lingao, Hainan (LG)	2008	Qi Li & Xiaodong Zheng	16	*Gafrarium dispar*	*Gafrarium dispar* A	HQ703088	BCC061-10
KL080304	Lingao, Hainan (LG)	2008	Qi Li & Xiaodong Zheng	16	*Gafrarium dispar*	*Gafrarium dispar* A	HQ703089	BCC062-10
KL080305	Lingao, Hainan (LG)	2008	Qi Li & Xiaodong Zheng	16	*Gafrarium dispar*	*Gafrarium dispar* A	HQ703090	BCC063-10
KW080201	Wenchang, Hainan (WC)	2008	Qi Li & Xiaodong Zheng	16	*Gafrarium dispar*	*Gafrarium dispar* A	HQ703091	BCC064-10
KW080401	Weizhou, Guangxi (WZ)	2008	Lingfeng Kong & Jun Chen	16	*Gafrarium dispar*	*Gafrarium dispar* A	HQ703092	BCC065-10
KS100401	Sanya, Hainan (SY)	2010	Lingfeng Kong & Jun Chen	16	*Gafrarium dispar*	*Gafrarium dispar* A	HQ703093	BCC066-10
KW100401	Weizhou, Guangxi (WZ)	2010	Lingfeng Kong & Jun Chen	19	*Gafrarium dispar*	*Gafrarium dispar* B	HQ703094	BCC067-10
QL080301	Lingao, Hainan (LG)	2008	Qi Li & Xiaodong Zheng	17	*Gafrarium divaricatum*	*Gafrarium divaricatum*	HQ703095	BCC068-10
QL080501	Lingao, Hainan (LG)	2008	Lingfeng Kong & Jun Chen	17	*Gafrarium divaricatum*	*Gafrarium divaricatum*	HQ703096	BCC069-10
QZ080411	Zhanjiang, Guangdong (ZJ)	2008	Daohai Chen	17	*Gafrarium divaricatum*	*Gafrarium divaricatum*	HQ703097	BCC070-10
QZ080412	Zhanjiang, Guangdong (ZJ)	2008	Daohai Chen	17	*Gafrarium divaricatum*	*Gafrarium divaricatum*	HQ703098	BCC071-10
QZ080413	Zhanjiang, Guangdong (ZJ)	2008	Daohai Chen	17	*Gafrarium divaricatum*	*Gafrarium divaricatum*	HQ703099	BCC072-10
QZ080414	Zhanjiang, Guangdong (ZJ)	2008	Daohai Chen	17	*Gafrarium divaricatum*	*Gafrarium divaricatum*	HQ703100	BCC073-10
QB080411	Beihai, Guangxi (BH)	2008	Lingfeng Kong & Jun Chen	17	*Gafrarium divaricatum*	*Gafrarium divaricatum*	HQ703101	BCC074-10

	QB080412	Beihai, Guangxi (BH)	2008	Lingfeng Kong & Jun Chen	17	*Gafrarium divaricatum*	*Gafrarium divaricatum*	HQ703102	BCC075-10
	QP090801	Pingtan, Fujian (PT)	2009	Qiaozhen Ke	17	*Gafrarium divaricatum*	*Gafrarium divaricatum*	HQ703103	BCC076-10
	QB100411	Beihai, Guangxi (BH)	2010	Lingfeng Kong & Jun Chen	17	*Gafrarium divaricatum*	*Gafrarium divaricatum*	HQ703104	BCC077-10
	QB100412	Beihai, Guangxi (BH)	2010	Lingfeng Kong & Jun Chen	17	*Gafrarium divaricatum*	*Gafrarium divaricatum*	HQ703105	BCC078-10
	QN100401	Naozhou, Guangdong (NZ)	2010	Lingfeng Kong & Jun Chen	17	*Gafrarium divaricatum*	*Gafrarium divaricatum*	HQ703106	BCC079-10
	MB080401	Beihai, Guangxi (BH)	2008	Lingfeng Kong & Jun Chen	7	*Circe scripta*	*Circe scripta* A	HQ703107	BCC080-10
	ML080501	Lingao, Hainan (LG)	2008	Lingfeng Kong & Jun Chen	7	*Circe scripta*	*Circe scripta* A	HQ703108	BCC081-10
	ML080502	Lingao, Hainan (LG)	2008	Lingfeng Kong & Jun Chen	7	*Circe scripta*	*Circe scripta* A	HQ703109	BCC082-10
	ML061201	Lingao, Hainan (LG)	2006	Rongxiang Gao	7	*Circe scripta*	*Circe scripta* A	HQ703110	BCC083-10
	MB100401	Beihai, Guangxi (BH)	2010	Lingfeng Kong & Jun Chen	7	*Circe scripta*	*Circe scripta* A	HQ703111	BCC084-10
	ML080301	Lingao, Hainan (LG)	2008	Qi Li & Xiaodong Zheng	7	*Circe scripta*	*Circe scripta* A	HQ703112	BCC085-10
	MS100401	Sanya, Hainan (SY)	2010	Lingfeng Kong & Jun Chen	8	*Circe scripta*	*Circe scripta* B	HQ703113	BCC086-10
	ML080201	Lingshui, Hainan (LS)	2008	Qi Li & Xiaodong Zheng	8	*Circe scripta*	*Circe scripta* B	HQ703114	BCC087-10
Cyclininae									
	QD071101	Zhoushan, Zhejiang (ZS)	2007	Xiaodong Zheng	11	*Cyclina sinensis*	*Cyclina sinensis*	HQ703115	BCC088-10
	QS071101	Shengsi, Zhejiang (SS)	2007	Xiaodong Zheng	11	*Cyclina sinensis*	*Cyclina sinensis*	HQ703116	BCC089-10
	QY080201	Yueqing, Zhejiang (YQ)	2008	Shuangshang Teng	11	*Cyclina sinensis*	*Cyclina sinensis*	HQ703117	BCC090-10
	QD080701	Dandong, Liaoning (DD)	2008	Hongtao Nie	11	*Cyclina sinensis*	*Cyclina sinensis*	HQ703118	BCC091-10
	QQ060801	Qinhuangdao, Hebei (QHD)	2006	Shikai Liu	11	*Cyclina sinensis*	*Cyclina sinensis*	HQ703119	BCC092-10
	QP080901	Panjin, Liaoning (PJ)	2008	Jie Bai	11	*Cyclina sinensis*	*Cyclina sinensis*	HQ703120	BCC093-10

	QD040401	Dongxing, Guangxi (DX)	2004	Ying Pan	11	*Cyclina sinensis*	*Cyclina sinensis*	HQ703121	BCC094-10
	QJ060301	Jimo, Shandong (JM)	2006	Qi Li	11	*Cyclina sinensis*	*Cyclina sinensis*	HQ703122	BCC095-10
	QL080901	Lianyugang, Jiangsu (LYG)	2008	Hongtao Nie	11	*Cyclina sinensis*	*Cyclina sinensis*	HQ703123	BCC096-10
	QL050801	Lvshun, Liaoning (LS)	2005	Yuming Zhao	11	*Cyclina sinensis*	*Cyclina sinensis*	HQ703124	BCC097-10
	QM050301	Maoming, Guangdong (MM)	2005	Ying Pan	11	*Cyclina sinensis*	*Cyclina sinensis*	HQ703125	BCC098-10
	QQ050101	Qidong, Jiangsu (QD)	2005	Yuming Zhao	11	*Cyclina sinensis*	*Cyclina sinensis*	HQ703126	BCC099-10
	QS080501	Sanya, Hainan (SY)	2008	Lingfeng Kong & Jun Chen	11	*Cyclina sinensis*	*Cyclina sinensis*	HQ703127	BCC100-10
	QT040401	Tanggu, Tianjing (TG)	2004	Yuming Zhao	11	*Cyclina sinensis*	*Cyclina sinensis*	HQ703128	BCC101-10
	QC040401	Changyi, Shandong (CY)	2004	Yuming Zhao	11	*Cyclina sinensis*	*Cyclina sinensis*	HQ703129	BCC102-10
	QX050501	Xiamen, Fujian (XM)	2005	Ying Pan	11	*Cyclina sinensis*	*Cyclina sinensis*	HQ703130	BCC103-10
	QX070801	Xiangshui, Jiangsu (XS)	2007	Xiaodong Zheng	11	*Cyclina sinensis*	*Cyclina sinensis*	HQ703131	BCC104-10
Dosiniinae									
	BF080901*	Fenshui, Shandong (FS)	2008	Hongtao Nie	124	*Phacosoma biscoticum*	*Phacosoma fibulum*	HQ703132	BCC105-10
	BC090801	Changle, Fujian (CL)	2009	Qiaozhen Ke	124	*Phacosoma biscoticum*	*Phacosoma fibulum*	HQ703133	BCC106-10
	XG080901	Ganyu, Jiangsu (GY)	2008	Hongtao Nie	124	*Phacosoma fibulum*	*Phacosoma fibulum*	HQ703134	BCC107-10
	XB080301	Beihai, Guangxi (BH)	2008	Lingfeng Kong & Jun Chen	124	*Phacosoma fibulum*	*Phacosoma fibulum*	HQ703135	BCC108-10
	XB100501	Beihai, Guangxi (BH)	2010	Lingfeng Kong & Jun Chen	124	*Phacosoma fibulum*	*Phacosoma fibulum*	HQ703136	BCC109-10
	RQ080801	Qingdao, Shandong (QD)	2008	Jun Chen & Yanwei Feng	27	*Phacosoma japonicum*	*Phacosoma japonicum*	HQ703137	BCC110-10
	RQ080802	Qingdao, Shandong (QD)	2008	Jun Chen & Yanwei Feng	27	*Phacosoma japonicum*	*Phacosoma japonicum*	HQ703138	BCC111-10
	SS061201	Sanya, Hainan (SY)	2006	Rongxiang Gao	125	*Phacosoma troscheli*	*Phacosoma troscheli*	HQ703139	BCC112-10

	SC090801	Changle, Fujian (CL)	2009	Qiaozhen Ke	125	*Phacosoma troscheli*	*Phacosoma troscheli*	HQ703140	BCC113-10
	SG080901	Ganyu, Jiangsu (GY)	2008	Hongtao Nie	14	*Dosinella* sp.	*Dosinella corrugata*	HQ703141	BCC114-10
	BG080911	Ganyu, Jiangsu (GY)	2009	Hongtao Nie	14	*Dosinella corrugata*	*Dosinella corrugata*	HQ703142	BCC115-10
	BP060701	Pulandian, Liaoning (PLD)	2006	Shikai Liu	14	*Dosinella corrugata*	*Dosinella corrugata*	HQ703143	BCC116-10
	BP060702	Pulandian, Liaoning (PLD)	2006	Shikai Liu	14	*Dosinella corrugata*	*Dosinella corrugata*	HQ703144	BCC117-10
	BP060703	Pulandian, Liaoning (PLD)	2006	Shikai Liu	14	*Dosinella corrugata*	*Dosinella corrugata*	HQ703145	BCC118-10
	BP060704*	Pulandian, Liaoning (PLD)	2006	Shikai Liu	14	*Dosinella corrugata*	*Dosinella corrugata*	HQ703146	BCC119-10
	BW080901*	Weihai, Shandong (WH)	2008	Yaosen Qian	14	*Dosinella corrugata**	*Dosinella corrugata*	HQ703147	BCC120-10
	JH060711	Haikou, Hainan (HK)	2006	Rongxiang Gao	13	*Dosinella angulosa**	*Dosinella angulosa*	HQ703148	BCC121-10
	JH060712	Haikou, Hainan (HK)	2006	Rongxiang Gao	13	*Dosinella angulosa*	*Dosinella angulosa*	HQ703149	BCC122-10
	JH060713	Haikou, Hainan (HK)	2006	Rongxiang Gao	13	*Dosinella angulosa*	*Dosinella angulosa*	HQ703150	BCC123-10
	JH060714	Haikou, Hainan (HK)	2006	Rongxiang Gao	13	*Dosinella angulosa*	*Dosinella angulosa*	HQ703151	BCC124-10
	SB080401	Beihai, Guangxi (BH)	2008	Lingfeng Kong & Jun Chen	4	Dosiniinae sp.	Dosiniinae sp.[#]	HQ703152	BCC125-10
	SS100411	Sanya, Hainan (SY)	2010	Lingfeng Kong & Jun Chen	4	Dosiniinae sp.	Dosiniinae sp.[#]	HQ703153	BCC126-10
	SB100401	Beihai, Guangxi (BH)	2010	Lingfeng Kong & Jun Chen	4	Dosiniinae sp.	Dosiniinae sp.[#]	HQ703154	BCC127-10
	SB100411*	Beihai, Guangxi (BH)	2010	Lingfeng Kong & Jun Chen	3	*Bonartemis histrio*	*Bonartemis histrio*	HQ703155	BCC128-10
	SB100412	Beihai, Guangxi (BH)	2010	Lingfeng Kong & Jun Chen	3	*Bonartemis histrio*	*Bonartemis histrio*	HQ703156	BCC129-10
	CS080201	Sanya, Hainan (SY)	2008	Xiaodong Zheng	6	*cf. Phacosoma* sp.	*cf. Phacosoma* sp.[#]	HQ703157	BCC130-10
Lioconchinae									
	AS100401	Sanya, Hainan (SY)	2010	Lingfeng Kong & Jun Chen	108	*Lioconcha annettae*	*Lioconcha annettae*[#]	HQ703158	BCC131-10

Meretricinae

	WL061201	Lingao, Hainan (LG)	2006	Rongxiang Gao	113	*Meretrix meretrix*	*Meretrix meretrix*	HQ703159	BCC132-10
	WL061202	Lingao, Hainan (LG)	2006	Rongxiang Gao	113	*Meretrix meretrix*	*Meretrix meretrix*	HQ703160	BCC133-10
	WL061203	Lingao, Hainan (LG)	2006	Rongxiang Gao	113	*Meretrix meretrix*	*Meretrix meretrix*	HQ703161	BCC134-10
	WB080301*	Beihai, Guangxi (BH)	2008	Lingfeng Kong & Jun Chen	113	*Meretrix meretrix*	*Meretrix meretrix*	HQ703162	BCC135-10
	WW061201	Weizhou, Guangxi (WZ)	2006	Rongxiang Gao	113	*Meretrix meretrix*	*Meretrix meretrix*	HQ703163	BCC136-10
	DP090401	Panjin, Liaoning (PJ)	2009	Jie Bai	28	*Meretrix petechialis*	*Meretrix petechialis* A	HQ703164	BCC137-10
	DP090402	Panjin, Liaoning (PJ)	2009	Jie Bai	28	*Meretrix petechialis*	*Meretrix petechialis* A	HQ703165	BCC138-10
	DG080901	Ganyu, Jiangsu (GY)	2008	Hongtao Nie	28	*Meretrix petechialis*	*Meretrix petechialis* A	HQ703166	BCC139-10
	DG080902*	Ganyu, Jiangsu (GY)	2008	Hongtao Nie	28	*Meretrix petechialis*	*Meretrix petechialis* A	HQ703167	BCC140-10
	DG080903	Ganyu, Jiangsu (GY)	2008	Hongtao Nie	28	*Meretrix petechialis*	*Meretrix petechialis* A	HQ703168	BCC141-10
	DN080501	Nanyang, Jiangsu (NT)	2008	Xiaodong Zheng	28	*Meretrix petechialis*	*Meretrix petechialis* A	HQ703169	BCC142-10
	DN080502*	Nanyang, Jiangsu (NT)	2008	Xiaodong Zheng	28	*Meretrix petechialis*	*Meretrix petechialis* A	HQ703170	BCC143-10
	DN080503*	Nanyang, Jiangsu (NT)	2008	Xiaodong Zheng	28	*Meretrix petechialis*	*Meretrix petechialis* A	HQ703171	BCC144-10
	DF081201	Fenshui, Shandong (FS)	2008	Hongtao Nie	28	*Meretrix petechialis*	*Meretrix petechialis* A	HQ703172	BCC145-10
	DS100401	Sanya, Hainan (SY)	2010	Lingfeng Kong & Jun Chen	72	*Meretrix petechialis*	*Meretrix petechialis* B	HQ703173	BCC146-10
	DX061201	Xiamen, Fujian (XM)	2006	Rongxiang Gao	72	*Meretrix petechialis*	*Meretrix petechialis* B	HQ703174	BCC147-10
	DX061202	Xiamen, Fujian (XM)	2006	Rongxiang Gao	72	*Meretrix petechialis*	*Meretrix petechialis* B	HQ703175	BCC148-10
	DZ080401	Zhanjiang, Guangdong (ZJ)	2008	Daohai Chen	72	*Meretrix petechialis*	*Meretrix petechialis* B	HQ703176	BCC149-10
	DZ080402	Zhanjiang, Guangdong (ZJ)	2008	Daohai Chen	72	*Meretrix petechialis*	*Meretrix petechialis* B	HQ703177	BCC150-10

DB100401	Beihai, Guangxi (BH)	2010	Lingfeng Kong & Jun Chen	72	*Meretrix petechialis*	*Meretrix petechialis* B	HQ703178	BCC151-10
DB100402	Beihai, Guangxi (BH)	2010	Lingfeng Kong & Jun Chen	72	*Meretrix petechialis*	*Meretrix petechialis* B	HQ703179	BCC152-10
DS100301	Shantou, Guangdong (ST)	2010	Qiaozhen Ke	72	*Meretrix petechialis*	*Meretrix petechialis* B	HQ703180	BCC153-10
DH091101	Haikou, Hainan (HK)	2009	Yuan Yuan	72	*Meretrix petechialis*	*Meretrix petechialis* B	HQ703181	BCC154-10
DH091102*	Haikou, Hainan (HK)	2009	Yuan Yuan	72	*Meretrix petechialis*	*Meretrix petechialis* B	HQ703182	BCC155-10
DL061201	Lingao, Hainan (LG)	2006	Rongxiang Gao	72	*Meretrix petechialis*	*Meretrix petechialis* B	HQ703183	BCC156-10
DY060901*	Yueqing, Zhejiang (WZ)	2006	Rongxiang Gao	72	*Meretrix petechialis*	*Meretrix petechialis* B	HQ703184	BCC157-10
DB080301	Beihai, Guangxi (BH)	2008	Qi Li & Xiaodong Zheng	72	*Meretrix petechialis*	*Meretrix petechialis* B	HQ703185	BCC158-10
DB080302*	Beihai, Guangxi (BH)	2008	Qi Li & Xiaodong Zheng	72	*Meretrix petechialis*	*Meretrix petechialis* B	HQ703186	BCC159-10
DY080201	Yangjiang, Guangdong (YJ)	2008	Daohai Chen	72	*Meretrix petechialis*	*Meretrix petechialis* B	HQ703187	BCC160-10
FH061201	Haikou, Hainan (HK)	2006	Rongxiang Gao	97	*Meretrix lamarckii*	*Meretrix lamarckii*	HQ703188	BCC161-10
FH061202	Haikou, Hainan (HK)	2006	Rongxiang Gao	97	*Meretrix lamarckii*	*Meretrix lamarckii*	HQ703189	BCC162-10
FH061203	Haikou, Hainan (HK)	2006	Rongxiang Gao	97	*Meretrix lamarckii*	*Meretrix lamarckii*	HQ703190	BCC163-10
FS100401	Sanya, Hainan (SY)	2010	Lingfeng Kong & Jun Chen	97	*Meretrix lamarckii*	*Meretrix lamarckii*	HQ703191	BCC164-10
QZ061201	Zhangpu, Fujian (ZP)	2006	Rongxiang Gao	98	*Meretrix lyrata*	*Meretrix lyrata*	HQ703192	BCC165-10
QZ061202	Zhangpu, Fujian (ZP)	2006	Rongxiang Gao	98	*Meretrix lyrata*	*Meretrix lyrata*	HQ703193	BCC166-10
QZ080401	Zhanjiang, Guangdong (ZJ)	2008	Daohai Chen	98	*Meretrix lyrata*	*Meretrix lyrata*	HQ703194	BCC167-10
QS061201	Sanya, Hainan (SY)	2006	Rongxiang Gao	98	*Meretrix lyrata*	*Meretrix lyrata*	HQ703195	BCC168-10
QS061202	Sanya, Hainan (SY)	2006	Rongxiang Gao	98	*Meretrix lyrata*	*Meretrix lyrata*	HQ703196	BCC169-10
QB080421	Beihai, Guangxi (BH)	2008	Lingfeng Kong & Jun Chen	98	*Meretrix lyrata*	*Meretrix lyrata*	HQ703197	BCC170-10

	QW080211	Wenchang, Hainan (WC)	2008	Qi Li & Xiaodong Zheng	98	*Meretrix lyrata*	*Meretrix lyrata*	HQ703198	BCC171-10
	CB080411	Beihai, Guangxi (BH)	2008	Lingfeng Kong & Jun Chen	114	*Meretrix* sp.	*Meretrix* sp.#	HQ703199	BCC172-10
	CH080401	Hepu, Guangxi (HP)	2008	Lingfeng Kong & Jun Chen	114	*Meretrix* sp.	*Meretrix* sp.#	HQ703200	BCC173-10
	CH080402	Hepu, Guangxi (HP)	2008	Lingfeng Kong & Jun Chen	114	*Meretrix* sp.	*Meretrix* sp.#	HQ703201	BCC174-10
	CH080403	Hepu, Guangxi (HP)	2008	Lingfeng Kong & Jun Chen	114	*Meretrix* sp.	*Meretrix* sp.#	HQ703202	BCC175-10
Pitarinae									
	XS080501	Sanya, Hainan (SY)	2008	Lingfeng Kong & Jun Chen	101	*Pitarina striatum*	*Pitarina striatum*	HQ703203	BCC176-10
	XS080502	Sanya, Hainan (SY)	2008	Lingfeng Kong & Jun Chen	101	*Pitarina striatum*	*Pitarina striatum*	HQ703204	BCC177-10
	XS080503	Sanya, Hainan (SY)	2008	Lingfeng Kong & Jun Chen	101	*Pitarina striatum*	*Pitarina striatum*	HQ703205	BCC178-10
	RS061201	Sanya, Hainan (SY)	2006	Rongxiang Gao	126	*Pitarina japonica*	*Pitarina japonica*	HQ703206	BCC179-10
	RL080501	Lingao, Hainan (LG)	2008	Lingfeng Kong & Jun Chen	126	*Pitarina japonica*	*Pitarina japonica*	HQ703207	BCC180-10
	SB100421	Beihai, Guangxi (BH)	2010	Lingfeng Kong & Jun Chen	127	*Pitarina* sp.	*Pitarina* sp.#	HQ703208	BCC181-10
	SQ080601	Qingdao, Shandong (QD)	2008	Jun Chen & Jingbo Wang	122	*Pelecyora isocardia*	*Pelecyora isocardia*	HQ703209	BCC182-10
	SF080901	Fenshui, Shandong (FS)	2008	Hongtao Nie	122	*Pelecyora isocardia*	*Pelecyora isocardia*	HQ703210	BCC183-10
Sunettinae									
	SN100601	Nanji, Zhejiang (NJ)	2010	Xiaodong Zheng & Lingfeng Kong	131	*Cyclosunetta* sp.	*Cyclosunetta* sp.#	HQ703211	BCC184-10
	SN100602	Nanji, Zhejiang (NJ)	2010	Xiaodong Zheng & Lingfeng Kong	131	*Cyclosunetta* sp.	*Cyclosunetta* sp.#	HQ703212	BCC185-10
	XF080601	Fenshui, Shandong (FS)	2008	Hongtao Nie	12	*Cyclosunetta menstrualis*	*Cyclosunetta menstrualis*	HQ703213	BCC186-10
	XF080602	Fenshui, Shandong (FS)	2008	Hongtao Nie	12	*Cyclosunetta menstrualis*	*Cyclosunetta menstrualis*	HQ703214	BCC187-10
	XF080603	Fenshui, Shandong (FS)	2008	Hongtao Nie	12	*Cyclosunetta menstrualis*	*Cyclosunetta menstrualis*	HQ703215	BCC188-10

	XF080604	Fenshui, Shandong (FS)	2008	Hongtao Nie	12	*Cyclosunetta menstrualis*	*Cyclosunetta menstrualis*	HQ703216	BCC189-10
Tapetinae									
	ZS080201	Sanya, Hainan (SY)	2008	Qi Li & Xiaodong Zheng	130	*Tapes literatus*	*Tapes literatus*	HQ703217	BCC190-10
	ZL080201	Lingshui, Hainan (LS)	2008	Qi Li & Xiaodong Zheng	130	*Tapes literatus* (*punctata* morph)	*Tapes literatus*	HQ703218	BCC191-10
	ZL080202	Lingshui, Hainan (LS)	2008	Qi Li & Xiaodong Zheng	130	*Tapes literatus* (*punctata* morph)	*Tapes literatus*	HQ703219	BCC192-10
	ZW080501	Wenchang, Hainan (WC)	2008	Lingfeng Kong & Jun Chen	130	*Tapes literatus* (*punctata* morph)	*Tapes literatus*	HQ703220	BCC193-10
	ZW080502	Wenchang, Hainan (WC)	2008	Lingfeng Kong & Jun Chen	130	*Tapes literatus*	*Tapes literatus*	HQ703221	BCC194-10
	ZS061201	Sanya, Hainan (SY)	2006	Rongxiang Gao	130	*Tapes literatus* (*punctata* morph)	*Tapes literatus*	HQ703222	BCC195-10
	ZS061202	Sanya, Hainan (SY)	2006	Rongxiang Gao	130	*Tapes literatus* (*punctata* morph)	*Tapes literatus*	HQ703223	BCC196-10
	DH080401	Hebu, Guangxi (HB)	2008	Lingfeng Kong & Jun Chen	94	*Tapes dorsatus*	*Tapes dorsatus*	HQ703224	BCC197-10
	DH080402	Hebu, Guangxi (HB)	2008	Lingfeng Kong & Jun Chen	94	*Tapes dorsatus*	*Tapes dorsatus*	HQ703225	BCC198-10
	DH080403	Hebu, Guangxi (HB)	2008	Lingfeng Kong & Jun Chen	94	*Tapes dorsatus*	*Tapes dorsatus*	HQ703226	BCC199-10
	DH080404	Hebu, Guangxi (HB)	2008	Lingfeng Kong & Jun Chen	94	*Tapes dorsatus*	*Tapes dorsatus*	HQ703227	BCC200-10
	DB080401*	Beihai, Guangxi (BH)	2008	Lingfeng Kong & Jun Chen	94	*Tapes dorsatus*	*Tapes dorsatus*	HQ703228	BCC201-10
	DB080201*	Beihai, Guangxi (BH)	2008	Qi Li & Xiaodong Zheng	94	*Tapes dorsatus*	*Tapes dorsatus*	HQ703229	BCC202-10
	DB080202*	Beihai, Guangxi (BH)	2008	Qi Li & Xiaodong Zheng	94	*Tapes dorsatus*	*Tapes dorsatus*	HQ703230	BCC203-10
	QP090811	Pingtan, Fujian (PT)	2009	Qiaozhen Ke	119	*Paphia sinuosa*	*Paphia sinuosa*	HQ703231	BCC204-10
	JP090801	Pingtan, Fujian (PT)	2009	Qiaozhen Ke	116	*Paphia gallus*	*Paphia gallus* A	HQ703232	BCC205-10
	JB080201	Baimajing, Hainan (BMJ)	2008	Qi Li & Xiaodong Zheng	96	*Paphia gallus*	*Paphia gallus* B	HQ703233	BCC206-10
	JB080202	Beihai, Guangxi (BH)	2008	Lingfeng Kong & Jun Chen	96	*Paphia gallus*	*Paphia gallus* B	HQ703234	BCC207-10

JB080203	Beihai, Guangxi (BH)	2008	Lingfeng Kong & Jun Chen	96	*Paphia gallus*	*Paphia gallus* B	HQ703235	BCC208-10
ZS061211	Sanya, Hainan (SY)	2006	Rongxiang Gao	120	*Paphia textile*	*Paphia textile*	HQ703236	BCC209-10
ZB080311	Beihai, Guangxi (BH)	2008	Lingfeng Kong & Jun Chen	120	*Paphia textile*	*Paphia textile*	HQ703237	BCC210-10
BW080911	Weihai, Shandong (WZ)	2008	Yaosen Qian	117	*Paphia papilionacea*	*Paphia papilionacea*	HQ703238	BCC211-10
BW080912	Weihai, Shandong (WZ)	2008	Yaosen Qian	117	*Paphia papilionacea*	*Paphia papilionacea*	HQ703239	BCC212-10
BW080913	Weihai, Shandong (WZ)	2008	Yaosen Qian	117	*Paphia papilionacea*	*Paphia papilionacea*	HQ703240	BCC213-10
BW080914	Weihai, Shandong (WZ)	2008	Yaosen Qian	117	*Paphia papilionacea*	*Paphia papilionacea*	HQ703241	BCC214-10
BQ080401	Qingdao, Shandong (QD)	2008	Jingbo Wang & Yanwei Feng	117	*Paphia papilionacea*	*Paphia papilionacea*	HQ703242	BCC215-10
BQ080402	Qingdao, Shandong (QD)	2008	Jingbo Wang & Yanwei Feng	117	*Paphia papilionacea*	*Paphia papilionacea*	HQ703243	BCC216-10
BY080201	Yangjiang, Guangdong (YJ)	2008	Daohai Chen	121	*Paphia undulata*	*Paphia undulata*	HQ703244	BCC217-10
BF080911	Fanggang, Guangxi (FG)	2008	Lingfeng Kong & Jun Chen	121	*Paphia undulata*	*Paphia undulata*	HQ703245	BCC218-10
BF080912	Fanggang, Guangxi (FG)	2008	Lingfeng Kong & Jun Chen	121	*Paphia undulata*	*Paphia undulata*	HQ703246	BCC219-10
BB080401*	Beihai, Guangxi (BH)	2008	Lingfeng Kong & Jun Chen	121	*Paphia undulata*	*Paphia undulata*	HQ703247	BCC220-10
BS100301	Shantou, Guangdong (ST)	2010	Qiaozhen Ke	121	*Paphia undulata*	*Paphia undulata*	HQ703248	BCC221-10
HB080301	Beihai, Guangxi (BH)	2008	Qi Li & Xiaodong Zheng	115	*Paphia amabilis* ※	*Paphia amabilis*	HQ703249	BCC222-10
HB080302	Beihai, Guangxi (BH)	2008	Qi Li & Xiaodong Zheng	115	*Paphia amabilis* ※	*Paphia amabilis*	HQ703250	BCC223-10
HB080303	Beihai, Guangxi (BH)	2008	Qi Li & Xiaodong Zheng	115	*Paphia amabilis*	*Paphia amabilis*	HQ703251	BCC224-10
HB080304	Beihai, Guangxi (BH)	2008	Qi Li & Xiaodong Zheng	115	*Paphia amabilis* ※	*Paphia amabilis*	HQ703252	BCC225-10
HB080305	Beihai, Guangxi (BH)	2008	Qi Li & Xiaodong Zheng	115	*Paphia amabilis*	*Paphia amabilis*	HQ703253	BCC226-10
HB080306	Beihai, Guangxi (BH)	2008	Qi Li & Xiaodong Zheng	115	*Paphia amabilis*	*Paphia amabilis*	HQ703254	BCC227-10

HB080307	Beihai, Guangxi (BH)	2008	Qi Li & Xiaodong Zheng	115	*Paphia amabilis* ※	*Paphia amabilis*	HQ703255	BCC228-10
HW061101	Wenchang, Hainan (WC)	2006	Rongxiang Gao	115	*Paphia amabilis* ※	*Paphia amabilis*	HQ703256	BCC229-10
HS080201	Sanya, Hainan (SY)	2008	Qi Li & Xiaodong Zheng	115	*Paphia amabilis* ※	*Paphia amabilis*	HQ703257	BCC230-10
HS080202	Sanya, Hainan (SY)	2008	Qi Li & Xiaodong Zheng	115	*Paphia amabilis* ※	*Paphia amabilis*	HQ703258	BCC231-10
HS080203	Sanya, Hainan (SY)	2008	Qi Li & Xiaodong Zheng	115	*Paphia amabilis* ※	*Paphia amabilis*	HQ703259	BCC232-10
HW080401	Weizhou, Guangxi (WZ)	2008	Lingfeng Kong & Jun Chen	115	*Paphia amabilis*	*Paphia amabilis*	HQ703260	BCC233-10
HW080402*	Weizhou, Guangxi (WZ)	2008	Lingfeng Kong & Jun Chen	115	*Paphia amabilis*	*Paphia amabilis*	HQ703261	BCC234-10
HW080403	Weizhou, Guangxi (WZ)	2008	Lingfeng Kong & Jun Chen	115	*Paphia amabilis*	*Paphia amabilis*	HQ703262	BCC235-10
HB080311	Beihai, Guangxi (BH)	2008	Lingfeng Kong & Jun Chen	118	*Paphia semirugata*	*Paphia semirugata*	HQ703263	BCC236-10
HB080312	Beihai, Guangxi (BH)	2008	Lingfeng Kong & Jun Chen	118	*Paphia semirugata*	*Paphia semirugata*	HQ703264	BCC237-10
HB080313	Beihai, Guangxi (BH)	2008	Lingfeng Kong & Jun Chen	118	*Paphia semirugata*	*Paphia semirugata*	HQ703265	BCC238-10
SH060501	Haiyang, Shangdong (HY)	2006	Ruihai Yu	110	*Macridiscus aequilatera*	*Macridiscus aequilatera*	HQ703266	BCC239-10
SH060502	Haiyang, Shangdong (HY)	2006	Ruihai Yu	110	*Macridiscus aequilatera*	*Macridiscus aequilatera*	HQ703267	BCC240-10
SH060503	Haiyang, Shangdong (HY)	2006	Ruihai Yu	110	*Macridiscus aequilatera*	*Macridiscus aequilatera*	HQ703268	BCC241-10
SH080801	Haiyang, Shangdong (HY)	2008	Qi Li	110	*Macridiscus aequilatera*	*Macridiscus aequilatera*	HQ703269	BCC242-10
SH080802	Haiyang, Shangdong (HY)	2008	Qi Li	110	*Macridiscus aequilatera*	*Macridiscus aequilatera*	HQ703270	BCC243-10
SH080803	Haiyang, Shangdong (HY)	2008	Qi Li	110	*Macridiscus aequilatera*	*Macridiscus aequilatera*	HQ703271	BCC244-10
SH080804*	Haiyang, Shangdong (HY)	2008	Qi Li	110	*Macridiscus aequilatera*	*Macridiscus aequilatera*	HQ703272	BCC245-10
HW080411	Weihai, Shandong (WZ)	2008	Qi Li	24	*Macridiscus melanaegis*	*Macridiscus melanaegis*	HQ703273	BCC246-10
HW080412	Weihai, Shandong (WZ)	2008	Qi Li	24	*Macridiscus melanaegis*	*Macridiscus melanaegis*	HQ703274	BCC247-10

BY080211	Yangjiang, Guangdong (YJ)	2008	Daohai Chen	109	*Macridiscus semicancellata*	*Macridiscus semicancellata*	HQ703275	BCC248-10
BB080201	Beihai, Guangxi (BH)	2008	Qi Li & Xiaodong Zheng	109	*Macridiscus semicancellata*	*Macridiscus semicancellata*	HQ703276	BCC249-10
BB080202	Beihai, Guangxi (BH)	2008	Qi Li & Xiaodong Zheng	109	*Macridiscus semicancellata*	*Macridiscus semicancellata*	HQ703277	BCC250-10
BB080203	Beihai, Guangxi (BH)	2008	Qi Li & Xiaodong Zheng	109	*Macridiscus semicancellata*	*Macridiscus semicancellata*	HQ703278	BCC251-10
BB080204*	Beihai, Guangxi (BH)	2008	Qi Li & Xiaodong Zheng	109	*Macridiscus semicancellata*	*Macridiscus semicancellata*	HQ703279	BCC252-10
BB100401	Beihai, Guangxi (BH)	2010	Lingfeng Kong & Jun Chen	109	*Macridiscus semicancellata*	*Macridiscus semicancellata*	HQ703280	BCC253-10
BP090801	Pingtan, Fujian (PT)	2009	Qiaozhen Ke	109	*Macridiscus semicancellata*	*Macridiscus semicancellata*	HQ703281	BCC254-10
RS080501	Sanya, Hainan (SY)	2008	Lingfeng Kong & Jun Chen	95	*Marcia japonica*	*Marcia japonica*	HQ703282	BCC255-10
RS080502	Sanya, Hainan (SY)	2008	Lingfeng Kong & Jun Chen	95	*Marcia japonica*	*Marcia japonica*	HQ703283	BCC256-10
RS080503	Sanya, Hainan (SY)	2008	Lingfeng Kong & Jun Chen	95	*Marcia japonica*	*Marcia japonica*	HQ703284	BCC257-10
RW061101	Wenchang, Hainan (WC)	2006	Rongxiang Gao	95	*Marcia japonica*	*Marcia japonica*	HQ703285	BCC258-10
RW061102	Wenchang, Hainan (WC)	2006	Rongxiang Gao	95	*Marcia japonica*	*Marcia japonica*	HQ703286	BCC259-10
LL061211	Lingshui, Hainan (LS)	2008	Qi Li & Xiaodong Zheng	111	*Marcia hiantina*	*Marcia hiantina*	HQ703287	BCC260-10
LZ080401	Zhanjiang, Guangdong (ZJ)	2008	Daohai Chen	111	*Marcia hiantina*	*Marcia hiantina*	HQ703288	BCC261-10
LM050401	Maoming, Guangdong (MM)	2005	Ying Pan	111	*Marcia hiantina*	*Marcia hiantina*	HQ703289	BCC262-10
LB080401	Beihai, Guangxi (BH)	2008	Lingfeng Kong & Jun Chen	111	*Marcia hiantina*	*Marcia hiantina*	HQ703290	BCC263-10
LB080402	Beihai, Guangxi (BH)	2008	Lingfeng Kong & Jun Chen	111	*Marcia hiantina*	*Marcia hiantina*	HQ703291	BCC264-10
LS080211*	Sanya, Hainan (SY)	2008	Qi Li & Xiaodong Zheng	111	*Marcia hiantina*	*Marcia hiantina*	HQ703292	BCC265-10
LS080212	Sanya, Hainan (SY)	2008	Qi Li & Xiaodong Zheng	111	*Marcia hiantina*	*Marcia hiantina*	HQ703293	BCC266-10
LS080213*	Sanya, Hainan (SY)	2008	Qi Li & Xiaodong Zheng	111	*Marcia hiantina*	*Marcia hiantina*	HQ703294	BCC267-10

SW061101	Wenchang, Hainan (WC)	2006	Rongxiang Gao	111	*Marcia* sp.1	*Marcia hiantina*	HQ703295	BCC268-10
LS080221	Sanya, Hainan (SY)	2008	Qi Li & Xiaodong Zheng	112	*Marcia* sp.2	*Marcia marmorata*	HQ703296	BCC269-10
LS080222	Sanya, Hainan (SY)	2008	Qi Li & Xiaodong Zheng	112	*Marcia marmorata*	*Marcia marmorata*	HQ703297	BCC270-10
LB080201	Beihai, Guangxi (BH)	2008	Qi Li & Xiaodong Zheng	112	*Marcia marmorata*	*Marcia marmorata*	HQ703298	BCC271-10
LB080202	Beihai, Guangxi (BH)	2008	Qi Li & Xiaodong Zheng	112	*Marcia marmorata*	*Marcia marmorata*	HQ703299	BCC272-10
LB080203*	Beihai, Guangxi (BH)	2008	Qi Li & Xiaodong Zheng	112	*Marcia marmorata*	*Marcia marmorata*	HQ703300	BCC273-10
LB080411	Beihai, Guangxi (BH)	2008	Lingfeng Kong & Jun Chen	112	*Marcia marmorata*	*Marcia marmorata*	HQ703301	BCC274-10
LB080412	Beihai, Guangxi (BH)	2008	Lingfeng Kong & Jun Chen	112	*Marcia marmorata*	*Marcia marmorata*	HQ703302	BCC275-10
SB080201	Baimajing, Hainan (BMJ)	2008	Qi Li & Xiaodong Zheng	112	*Marcia* sp.2	*Marcia marmorata*	HQ703303	BCC276-10
FJ080801*	Jimo, Shandong (JM)	2008	Lingfeng Kong & Jun Chen	21	*Ruditapes philippinarum*	*Ruditapes philippinarum*	HQ703304	BCC277-10
FR080901	Rongcheng, Shandong (RC)	2008	Lingfeng Kong & Jun Chen	21	*Ruditapes philippinarum*	*Ruditapes philippinarum*	HQ703305	BCC278-10
FZ061201	Zhangpu, Fujian (ZP)	2006	Rongxiang Gao	21	*Ruditapes philippinarum*	*Ruditapes philippinarum*	HQ703306	BCC279-10
FZ061202	Zhangpu, Fujian (ZP)	2006	Rongxiang Gao	21	*Ruditapes philippinarum*	*Ruditapes philippinarum*	HQ703307	BCC280-10
FY081101	Shenzhen, Guangdong (SZ)	2008	Wenguang Liu	21	*Ruditapes philippinarum*	*Ruditapes philippinarum*	HQ703308	BCC281-10
FG080901	Ganyu, Jiangsu (GY)	2008	Hongtao Nie	21	*Ruditapes philippinarum*	*Ruditapes philippinarum*	HQ703309	BCC282-10
FN070601*	Nanji, Zhejiang (NJ)	2007	Xiaodong Zheng	21	*Ruditapes philippinarum*	*Ruditapes philippinarum*	HQ703310	BCC283-10
FB080201	Baimajing, Hainan (BMJ)	2008	Qi Li & Xiaodong Zheng	21	*Ruditapes philippinarum*	*Ruditapes philippinarum*	HQ703311	BCC284-10
ZB080401	Beihai, Guangxi (BH)	2007	Lingfeng Kong & Jun Chen	55	*Ruditapes variegata*	*Ruditapes variegata*	HQ703312	BCC285-10
ZB080402	Beihai, Guangxi (BH)	2008	Lingfeng Kong & Jun Chen	55	*Ruditapes variegata*	*Ruditapes variegata*	HQ703313	BCC286-10
ZW080401	Weizhou, Guangxi (WZ)	2008	Lingfeng Kong & Jun Chen	55	*Ruditapes variegata*	*Ruditapes variegata*	HQ703314	BCC287-10

	ZS080211	Sanya, Hainan (SY)	2008	Qi Li & Xiaodong Zheng	55	*Ruditapes variegata*	*Ruditapes variegata*	HQ703315	BCC288-10
	ZS080212	Sanya, Hainan (SY)	2008	Qi Li & Xiaodong Zheng	55	*Ruditapes variegata*	*Ruditapes variegata*	HQ703316	BCC289-10
	ZW100401	Weizhou, Guangxi (WZ)	2010	Lingfeng Kong & Jun Chen	55	*Ruditapes variegata*	*Ruditapes variegata*	HQ703317	BCC290-10
Venerinae									
	ZS080501	Sanya, Hainan (SY)	2008	Lingfeng Kong & Jun Chen	102	*Periglypta puerpera*	*Periglypta puerpera* A	HQ703318	BCC291-10
	ZS080502	Sanya, Hainan (SY)	2008	Lingfeng Kong & Jun Chen	102	*Periglypta puerpera*	*Periglypta puerpera* A	HQ703319	BCC292-10
	ZS080503	Sanya, Hainan (SY)	2008	Lingfeng Kong & Jun Chen	102	*Periglypta puerpera*	*Periglypta puerpera* A	HQ703320	BCC293-10
	ZS080504	Sanya, Hainan (SY)	2008	Lingfeng Kong & Jun Chen	102	*Periglypta puerpera*	*Periglypta puerpera* A	HQ703321	BCC294-10
	ZL080211	Lingshui, Hainan (LS)	2008	Xiaodong Zheng & Qi Li	102	*Periglypta puerpera*	*Periglypta puerpera* A	HQ703322	BCC295-10
	ZL080301	Wenchang, Hainan (WC)	2008	Xiaodong Zheng & Qi Li	102	*Periglypta puerpera*	*Periglypta puerpera* A	HQ703323	BCC296-10
	ZL080302	Wenchang, Hainan (WC)	2008	Xiaodong Zheng & Qi Li	102	*Periglypta puerpera*	*Periglypta puerpera* A	HQ703324	BCC297-10
	ZQ100401	Qionghai, Hainan (QH)	2010	Lingfeng Kong & Jun Chen	102	*Periglypta puerpera*	*Periglypta puerpera* A	HQ703325	BCC298-10
	ZQ100402	Qionghai, Hainan (QH)	2010	Lingfeng Kong & Jun Chen	102	*Periglypta puerpera*	*Periglypta puerpera* A	HQ703326	BCC299-10
	ZQ100403	Qionghai, Hainan (QH)	2010	Lingfeng Kong & Jun Chen	102	*Periglypta puerpera*	*Periglypta puerpera* A	HQ703327	BCC300-10
	ZH100401	Haikou, Hainan (HK)	2010	Lingfeng Kong & Jun Chen	102	*Periglypta puerpera*	*Periglypta puerpera* A	HQ703328	BCC301-10
	ZS080221	Sanya, Hainan (SY)	2008	Xiaodong Zheng & Qi Li	82	*Periglypta puerpera*	*Periglypta puerpera* B	HQ703329	BCC302-10
	BB080411	Beihai, Guangxi (BH)	2008	Lingfeng Kong & Jun Chen	123	*Periglypta chemnitzii*	*Periglypta chemnitzii*	HQ703330	BCC303-10
	BB080412	Beihai, Guangxi (BH)	2008	Lingfeng Kong & Jun Chen	123	*Periglypta chemnitzii*	*Periglypta chemnitzii*	HQ703331	BCC304-10
	BB080413	Beihai, Guangxi (BH)	2008	Lingfeng Kong & Jun Chen	123	*Periglypta chemnitzii*	*Periglypta chemnitzii*	HQ703332	BCC305-10
	BB080414	Beihai, Guangxi (BH)	2008	Lingfeng Kong & Jun Chen	123	*Periglypta chemnitzii*	*Periglypta chemnitzii*	HQ703333	BCC306-10

DS080201	Sanya, Hainan (SY)	2008	Xiaodong Zheng & Qi Li	2	*Antigona lamellaris*	*Antigona lamellaris*	HQ703334	BCC307-10
DS080202	Sanya, Hainan (SY)	2008	Xiaodong Zheng & Qi Li	2	*Antigona lamellaris*	*Antigona lamellaris*	HQ703335	BCC308-10
DS080203	Sanya, Hainan (SY)	2008	Xiaodong Zheng & Qi Li	2	*Antigona lamellaris*	*Antigona lamellaris*	HQ703336	BCC309-10
DB080411	Beihai, Guangxi (BH)	2008	Lingfeng Kong & Jun Chen	2	*Antigona lamellaris*	*Antigona lamellaris*	HQ703337	BCC310-10
DB080412	Beihai, Guangxi (BH)	2008	Lingfeng Kong & Jun Chen	2	*Antigona lamellaris*	*Antigona lamellaris*	HQ703338	BCC311-10
DB080413	Beihai, Guangxi (BH)	2008	Lingfeng Kong & Jun Chen	2	*Antigona lamellaris*	*Antigona lamellaris*	HQ703339	BCC312-10
DB080414	Beihai, Guangxi (BH)	2008	Lingfeng Kong & Jun Chen	2	*Antigona lamellaris*	*Antigona lamellaris*	HQ703340	BCC313-10
DB080415*	Beihai, Guangxi (BH)	2008	Lingfeng Kong & Jun Chen	2	*Antigona lamellaris*	*Antigona lamellaris*	HQ703341	BCC314-10
DL080301	Lingao, Hainan (LG)	2008	Xiaodong Zheng & Qi Li	107	*Globivenus toreuma*	*Globivenus toreuma*	HQ703342	BCC315-10

[§]: cut-off value is 4%

*: juvenile specimen

[#]: first record from China or new to science

N/A: not applicable

—: unable to match any records in BOLD database

[※]: individual of *Paphia schnelliana* Zhuang (2001) (non Dunker, 1862)
