# Supplementary material for: How DNA Barcodes Complement Taxonomy and Explore Species Diversity: The Case Study of a Poorly Understood Marine Fauna
Source: PLoS One. 2011 Jun 16;6(6):e21326. doi: 10.1371/journal.pone.0021326 (PMC3116896; doi:10.1371/journal.pone.0021326)
Supplement: Figure S1 — A neighbour-joining tree of 622 COI sequence from venerid species sampled by ourselves and obtained in BOLD, using K2P distances. Numbers near the nodes indicate NJ bootstrap support. Species names and GenBank accession numbers are given at branch tips. (PDF) [file pone.0021326.s003.pdf]

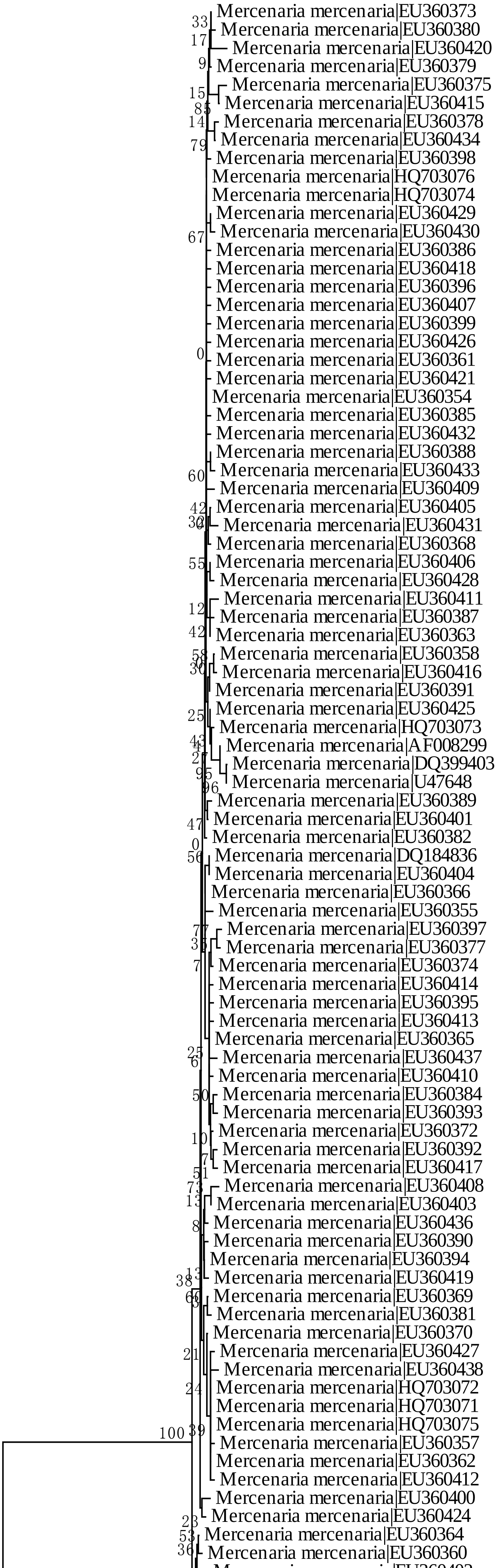
Mercenaria mercenaria|EU360373
Mercenaria mercenaria|EU360380
Mercenaria mercenaria|EU360420
Mercenaria mercenaria|EU360379
Mercenaria mercenaria|EU360375
Mercenaria mercenaria|EU360415
Mercenaria mercenaria|EU360378
Mercenaria mercenaria|EU360434
Mercenaria mercenaria|EU360398
Mercenaria mercenaria|HQ703076
Mercenaria mercenaria|HQ703074
Mercenaria mercenaria|EU360429
Mercenaria mercenaria|EU360430
Mercenaria mercenaria|EU360386
Mercenaria mercenaria|EU360418
Mercenaria mercenaria|EU360396
Mercenaria mercenaria|EU360407
Mercenaria mercenaria|EU360399
Mercenaria mercenaria|EU360426
Mercenaria mercenaria|EU360361
Mercenaria mercenaria|EU360421
Mercenaria mercenaria|EU360354
Mercenaria mercenaria|EU360385
Mercenaria mercenaria|EU360432
Mercenaria mercenaria|EU360388
Mercenaria mercenaria|EU360433
Mercenaria mercenaria|EU360409
Mercenaria mercenaria|EU360405
Mercenaria mercenaria|EU360431
Mercenaria mercenaria|EU360368
Mercenaria mercenaria|EU360406
Mercenaria mercenaria|EU360428
Mercenaria mercenaria|EU360411
Mercenaria mercenaria|EU360387
Mercenaria mercenaria|EU360363
Mercenaria mercenaria|EU360358
Mercenaria mercenaria|EU360416
Mercenaria mercenaria|EU360391
Mercenaria mercenaria|EU360425
Mercenaria mercenaria|HQ703073
Mercenaria mercenaria|AF008299
Mercenaria mercenaria|DQ399403
Mercenaria mercenaria|U47648
Mercenaria mercenaria|EU360389
Mercenaria mercenaria|EU360401
Mercenaria mercenaria|EU360382
Mercenaria mercenaria|DQ184836
Mercenaria mercenaria|EU360404
Mercenaria mercenaria|EU360366
Mercenaria mercenaria|EU360355
Mercenaria mercenaria|EU360397
Mercenaria mercenaria|EU360377
Mercenaria mercenaria|EU360374
Mercenaria mercenaria|EU360414
Mercenaria mercenaria|EU360395
Mercenaria mercenaria|EU360413
Mercenaria mercenaria|EU360365
Mercenaria mercenaria|EU360437
Mercenaria mercenaria|EU360410
Mercenaria mercenaria|EU360384
Mercenaria mercenaria|EU360393
Mercenaria mercenaria|EU360372
Mercenaria mercenaria|EU360392
Mercenaria mercenaria|EU360417
Mercenaria mercenaria|EU360408
Mercenaria mercenaria|EU360403
Mercenaria mercenaria|EU360436
Mercenaria mercenaria|EU360390
Mercenaria mercenaria|EU360394
Mercenaria mercenaria|EU360419
Mercenaria mercenaria|EU360369
Mercenaria mercenaria|EU360381
Mercenaria mercenaria|EU360370
Mercenaria mercenaria|EU360427
Mercenaria mercenaria|EU360438
Mercenaria mercenaria|HQ703072
Mercenaria mercenaria|HQ703071
Mercenaria mercenaria|HQ703075
Mercenaria mercenaria|EU360357
Mercenaria mercenaria|EU360362
Mercenaria mercenaria|EU360412
Mercenaria mercenaria|EU360400
Mercenaria mercenaria|EU360424
Mercenaria mercenaria|EU360364
Mercenaria mercenaria|EU360360
100

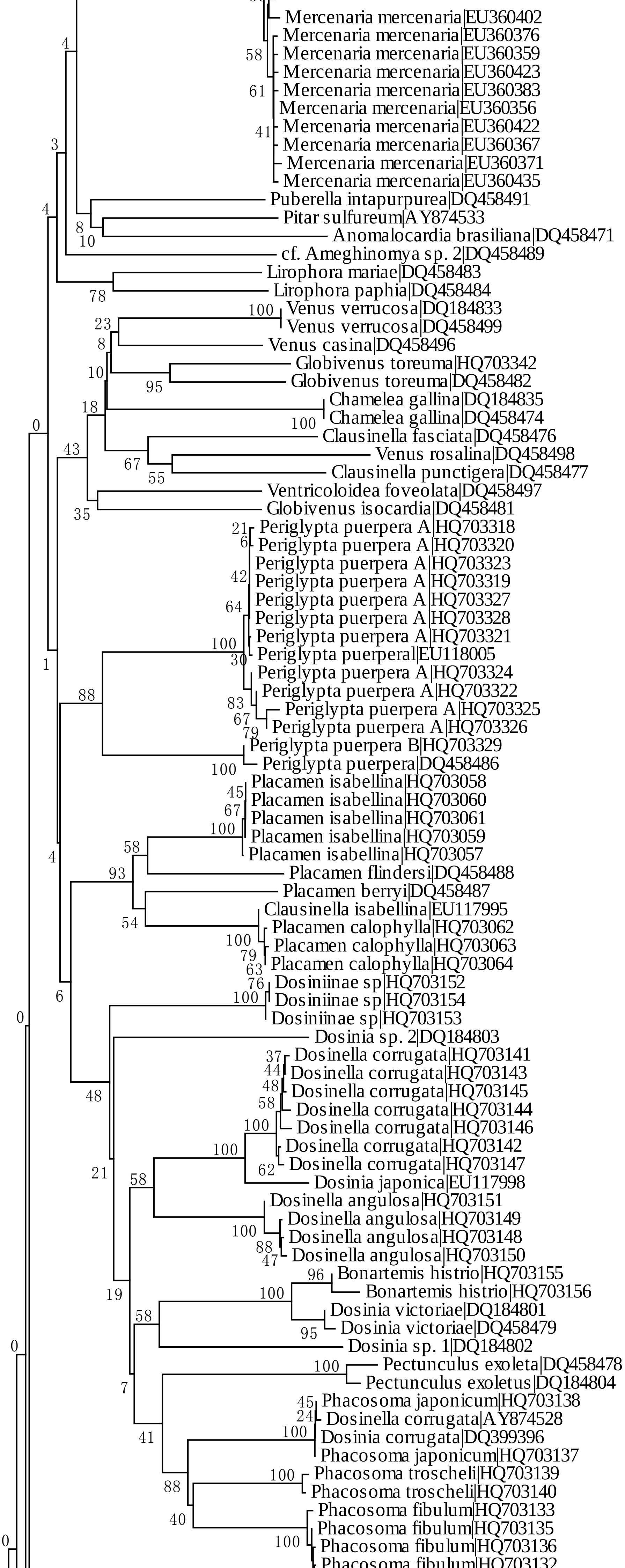
Mercenaria mercenaria|EU360402
Mercenaria mercenaria|EU360376
Mercenaria mercenaria|EU360359
Mercenaria mercenaria|EU360423
Mercenaria mercenaria|EU360383
Mercenaria mercenaria|EU360356
Mercenaria mercenaria|EU360422
Mercenaria mercenaria|EU360367
Mercenaria mercenaria|EU360371
Mercenaria mercenaria|EU360435
Puberella intapurpurea|DQ458491
Pitar sulfureum|AY874533
Anomalocardia brasiliana|DQ458471
cf. Ameghinomya sp. 2|DQ458489
Lirophora mariae|DQ458483
Lirophora paphia|DQ458484
Venus verrucosa|DQ184833
Venus verrucosa|DQ458499
Venus casina|DQ458496
Globivenus toreuma|HQ703342
Globivenus toreuma|DQ458482
Chamelea gallina|DQ184835
Chamelea gallina|DQ458474
Clausinella fasciata|DQ458476
Venus rosalina|DQ458498
Clausinella punctigera|DQ458477
Ventricoloidea foveolata|DQ458497
Globivenus isocardia|DQ458481
Periglypta puerpera A|HQ703318
Periglypta puerpera A|HQ703320
Periglypta puerpera A|HQ703323
Periglypta puerpera A|HQ703319
Periglypta puerpera A|HQ703327
Periglypta puerpera A|HQ703328
Periglypta puerpera A|HQ703321
Periglypta puerperal|EU118005
Periglypta puerpera A|HQ703324
Periglypta puerpera A|HQ703322
Periglypta puerpera A|HQ703325
Periglypta puerpera A|HQ703326
Periglypta puerpera B|HQ703329
Periglypta puerpera|DQ458486
Placamen isabellina|HQ703058
Placamen isabellina|HQ703060
Placamen isabellina|HQ703061
Placamen isabellina|HQ703059
Placamen isabellina|HQ703057
Placamen flindersi|DQ458488
Placamen berryi|DQ458487
Clausinella isabellina|EU117995
Placamen calophylla|HQ703062
Placamen calophylla|HQ703063
Placamen calophylla|HQ703064
Dosiniinae sp|HQ703152
Dosiniinae sp|HQ703154
Dosiniinae sp|HQ703153
Dosinia sp. 2|DQ184803
Dosinella corrugata|HQ703141
Dosinella corrugata|HQ703143
Dosinella corrugata|HQ703145
Dosinella corrugata|HQ703144
Dosinella corrugata|HQ703146
Dosinella corrugata|HQ703142
Dosinella corrugata|HQ703147
Dosinia japonica|EU117998
Dosinella angulosa|HQ703151
Dosinella angulosa|HQ703149
Dosinella angulosa|HQ703148
Dosinella angulosa|HQ703150
Bonartemis histrio|HQ703155
Bonartemis histrio|HQ703156
Dosinia victoriae|DQ184801
Dosinia victoriae|DQ458479
Dosinia sp. 1|DQ184802
Pectunculus exoleta|DQ458478
Pectunculus exoletus|DQ184804
Phacosoma japonicum|HQ703138
Dosinella corrugata|AY874528
Dosinia corrugata|DQ399396
Phacosoma japonicum|HQ703137
Phacosoma troscheli|HQ703139
Phacosoma troscheli|HQ703140
Phacosoma fibulum|HQ703133
Phacosoma fibulum|HQ703135
Phacosoma fibulum|HQ703136
Phacosoma fibulum|HQ703132

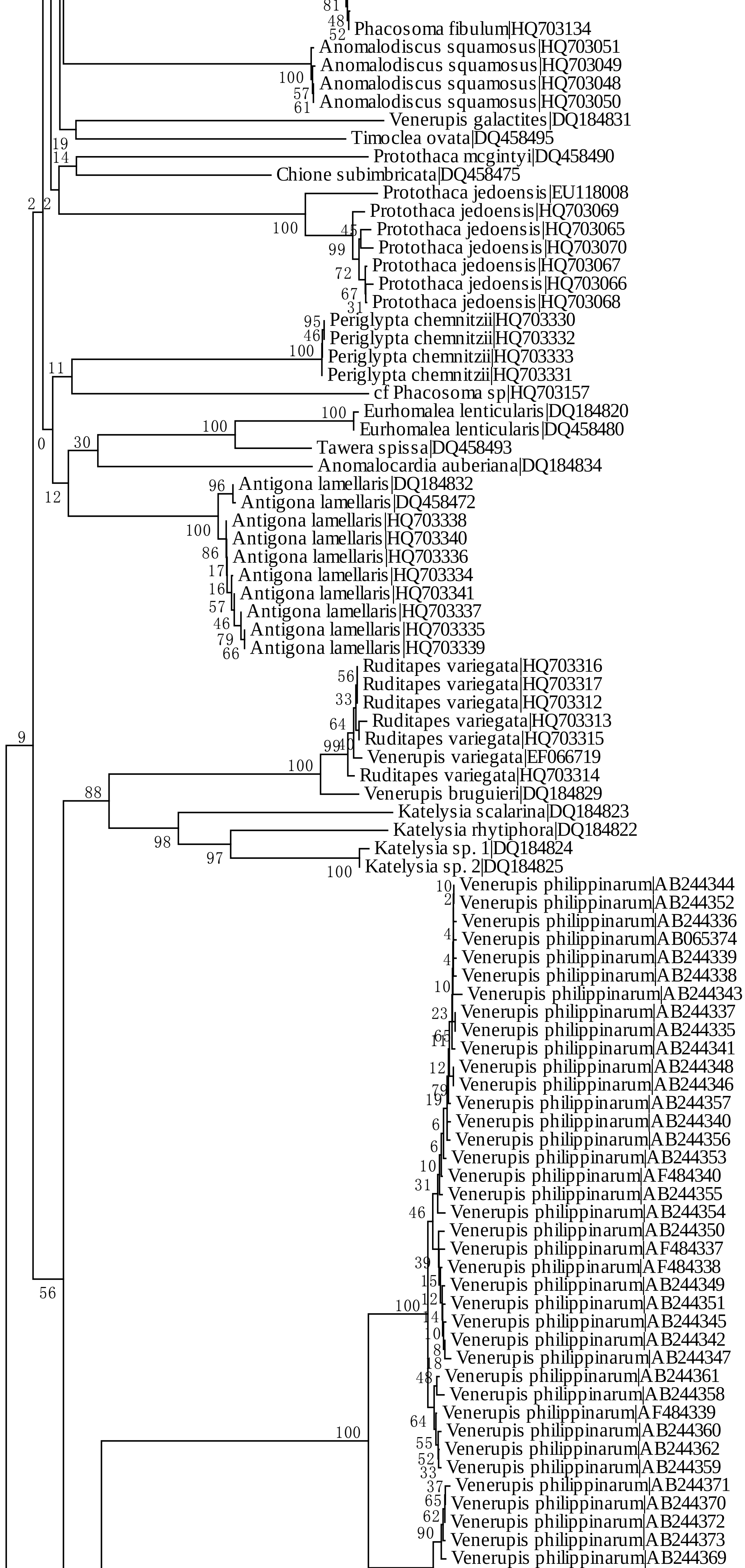
Phacosoma fibulum|HQ703134
Anomalodiscus squamosus|HQ703051
Anomalodiscus squamosus|HQ703049
Anomalodiscus squamosus|HQ703048
Anomalodiscus squamosus|HQ703050
Venerupis galactites|DQ184831
Timoclea ovata|DQ458495
Protothaca mcgintyi|DQ458490
Chione subimbricata|DQ458475
Protothaca jedoensis|EU118008
Protothaca jedoensis|HQ703069
Protothaca jedoensis|HQ703065
Protothaca jedoensis|HQ703070
Protothaca jedoensis|HQ703067
Protothaca jedoensis|HQ703066
Protothaca jedoensis|HQ703068
Periglypta chemnitzii|HQ703330
Periglypta chemnitzii|HQ703332
Periglypta chemnitzii|HQ703333
Periglypta chemnitzii|HQ703331
cf Phacosoma sp|HQ703157
Eurhomalea lenticularis|DQ184820
Eurhomalea lenticularis|DQ458480
Tawera spissa|DQ458493
Anomalocardia auberiana|DQ184834
Antigona lamellaris|DQ184832
Antigona lamellaris|DQ458472
Antigona lamellaris|HQ703338
Antigona lamellaris|HQ703340
Antigona lamellaris|HQ703336
Antigona lamellaris|HQ703334
Antigona lamellaris|HQ703341
Antigona lamellaris|HQ703337
Antigona lamellaris|HQ703335
Antigona lamellaris|HQ703339
Ruditapes variegata|HQ703316
Ruditapes variegata|HQ703317
Ruditapes variegata|HQ703312
Ruditapes variegata|HQ703313
Ruditapes variegata|HQ703315
Venerupis variegata|EF066719
Ruditapes variegata|HQ703314
Venerupis bruguieri|DQ184829
Katelysia scalarina|DQ184823
Katelysia rhytiphora|DQ184822
Katelysia sp. 1|DQ184824
Katelysia sp. 2|DQ184825
Venerupis philippinarum|AB244344
Venerupis philippinarum|AB244352
Venerupis philippinarum|AB244336
Venerupis philippinarum|AB065374
Venerupis philippinarum|AB244339
Venerupis philippinarum|AB244338
Venerupis philippinarum|AB244343
Venerupis philippinarum|AB244337
Venerupis philippinarum|AB244335
Venerupis philippinarum|AB244341
Venerupis philippinarum|AB244348
Venerupis philippinarum|AB244346
Venerupis philippinarum|AB244357
Venerupis philippinarum|AB244340
Venerupis philippinarum|AB244356
Venerupis philippinarum|AB244353
Venerupis philippinarum|AF484340
Venerupis philippinarum|AB244355
Venerupis philippinarum|AB244354
Venerupis philippinarum|AB244350
Venerupis philippinarum|AF484337
Venerupis philippinarum|AF484338
Venerupis philippinarum|AB244349
Venerupis philippinarum|AB244351
Venerupis philippinarum|AB244345
Venerupis philippinarum|AB244342
Venerupis philippinarum|AB244347
Venerupis philippinarum|AB244361
Venerupis philippinarum|AB244358
Venerupis philippinarum|AF484339
Venerupis philippinarum|AB244360
Venerupis philippinarum|AB244362
Venerupis philippinarum|AB244359
Venerupis philippinarum|AB244371
Venerupis philippinarum|AB244370
Venerupis philippinarum|AB244372
Venerupis philippinarum|AB244373
Venerupis philippinarum|AB244369

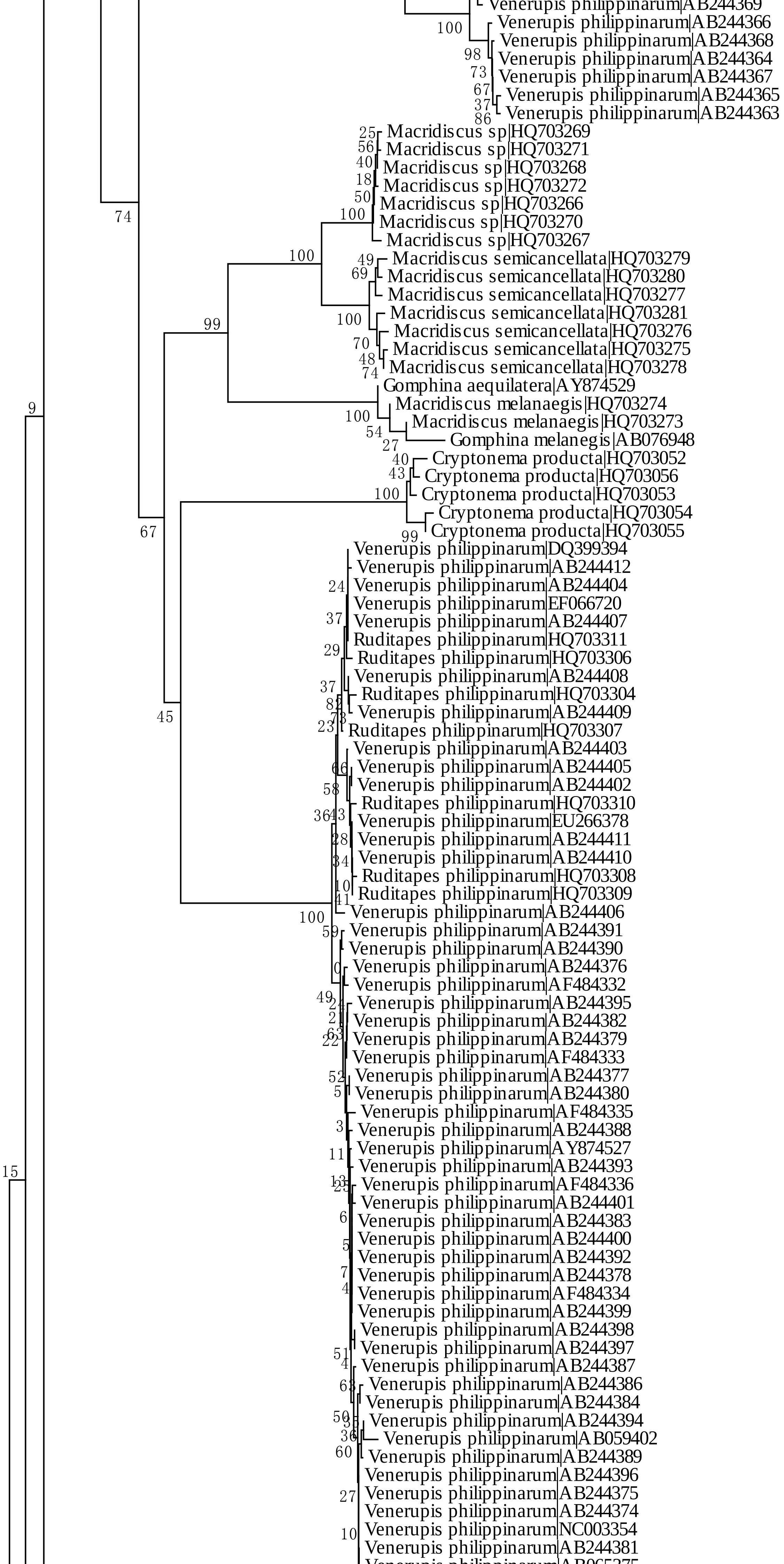
Venerupis philippinarum|AB244369
Venerupis philippinarum|AB244366
Venerupis philippinarum|AB244368
Venerupis philippinarum|AB244364
Venerupis philippinarum|AB244367
Venerupis philippinarum|AB244365
Venerupis philippinarum|AB244363
Macridiscus sp|HQ703269
Macridiscus sp|HQ703271
Macridiscus sp|HQ703268
Macridiscus sp|HQ703272
Macridiscus sp|HQ703266
Macridiscus sp|HQ703270
Macridiscus sp|HQ703267
Macridiscus semicancellata|HQ703279
Macridiscus semicancellata|HQ703280
Macridiscus semicancellata|HQ703277
Macridiscus semicancellata|HQ703281
Macridiscus semicancellata|HQ703276
Macridiscus semicancellata|HQ703275
Macridiscus semicancellata|HQ703278
Gomphina aequilatera|AY874529
Macridiscus melanaegis|HQ703274
Macridiscus melanaegis|HQ703273
Gomphina melanegis|AB076948
Cryptonema producta|HQ703052
Cryptonema producta|HQ703056
Cryptonema producta|HQ703053
Cryptonema producta|HQ703054
Cryptonema producta|HQ703055
Venerupis philippinarum|DQ399394
Venerupis philippinarum|AB244412
Venerupis philippinarum|AB244404
Venerupis philippinarum|EF066720
Venerupis philippinarum|AB244407
Ruditapes philippinarum|HQ703311
Ruditapes philippinarum|HQ703306
Venerupis philippinarum|AB244408
Ruditapes philippinarum|HQ703304
Venerupis philippinarum|AB244409
Ruditapes philippinarum|HQ703307
Venerupis philippinarum|AB244403
Venerupis philippinarum|AB244405
Venerupis philippinarum|AB244402
Ruditapes philippinarum|HQ703310
Venerupis philippinarum|EU266378
Venerupis philippinarum|AB244411
Venerupis philippinarum|AB244410
Ruditapes philippinarum|HQ703308
Ruditapes philippinarum|HQ703309
Venerupis philippinarum|AB244406
Venerupis philippinarum|AB244391
Venerupis philippinarum|AB244390
Venerupis philippinarum|AB244376
Venerupis philippinarum|AF484332
Venerupis philippinarum|AB244395
Venerupis philippinarum|AB244382
Venerupis philippinarum|AB244379
Venerupis philippinarum|AF484333
Venerupis philippinarum|AB244377
Venerupis philippinarum|AB244380
Venerupis philippinarum|AF484335
Venerupis philippinarum|AB244388
Venerupis philippinarum|AY874527
Venerupis philippinarum|AB244393
Venerupis philippinarum|AF484336
Venerupis philippinarum|AB244401
Venerupis philippinarum|AB244383
Venerupis philippinarum|AB244400
Venerupis philippinarum|AB244392
Venerupis philippinarum|AB244378
Venerupis philippinarum|AF484334
Venerupis philippinarum|AB244399
Venerupis philippinarum|AB244398
Venerupis philippinarum|AB244397
Venerupis philippinarum|AB244387
Venerupis philippinarum|AB244386
Venerupis philippinarum|AB244384
Venerupis philippinarum|AB244394
Venerupis philippinarum|AB059402
Venerupis philippinarum|AB244389
Venerupis philippinarum|AB244396
Venerupis philippinarum|AB244375
Venerupis philippinarum|AB244374
Venerupis philippinarum|NC003354
Venerupis philippinarum|AB244381

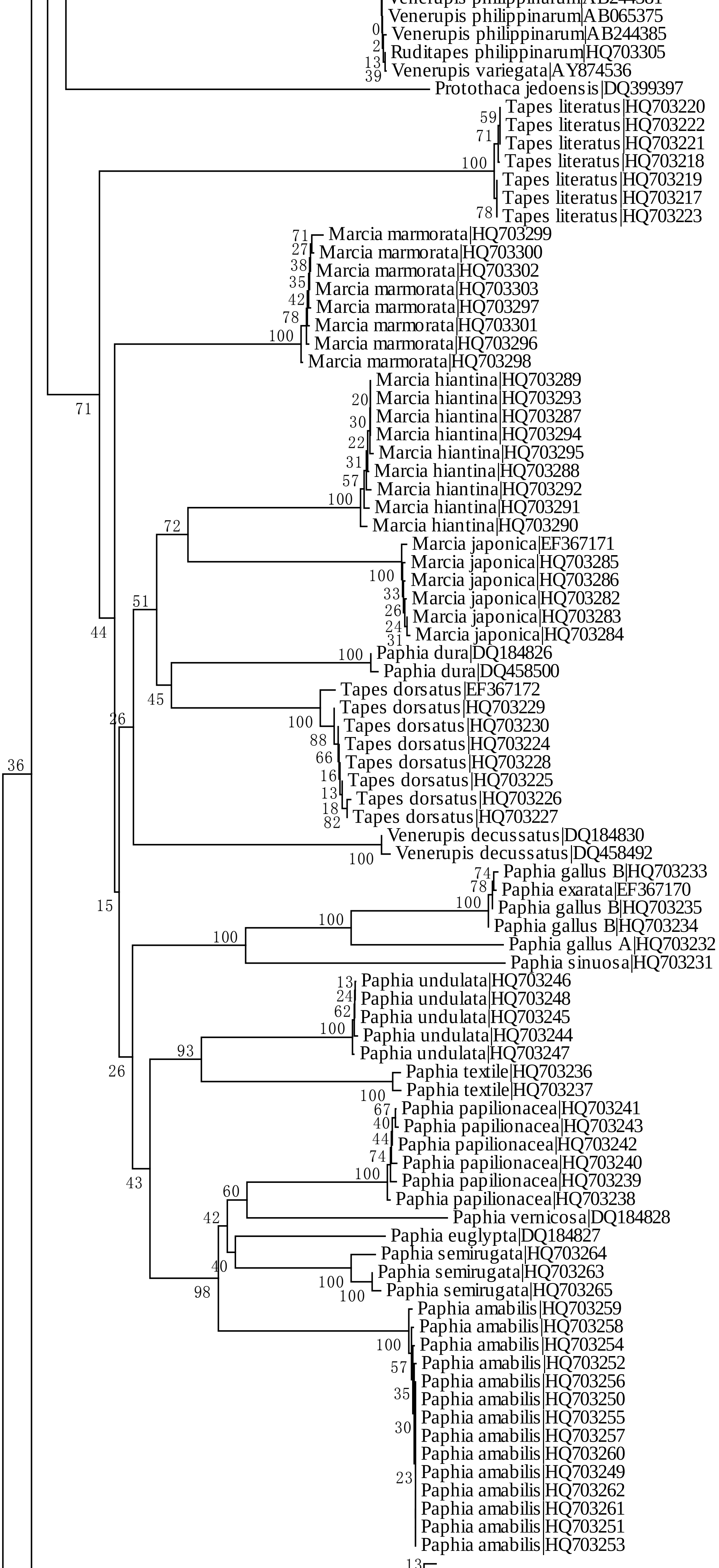
Venerupis philippinarum|AB065375
Venerupis philippinarum|AB244385
Ruditapes philippinarum|HQ703305
Venerupis variegata|AY874536
Protothaca jedoensis|DQ399397
Tapes literatus|HQ703220
Tapes literatus|HQ703222
Tapes literatus|HQ703221
Tapes literatus|HQ703218
Tapes literatus|HQ703219
Tapes literatus|HQ703217
Tapes literatus|HQ703223
Marcia marmorata|HQ703299
Marcia marmorata|HQ703300
Marcia marmorata|HQ703302
Marcia marmorata|HQ703303
Marcia marmorata|HQ703297
Marcia marmorata|HQ703301
Marcia marmorata|HQ703296
Marcia marmorata|HQ703298
Marcia hiantina|HQ703289
Marcia hiantina|HQ703293
Marcia hiantina|HQ703287
Marcia hiantina|HQ703294
Marcia hiantina|HQ703295
Marcia hiantina|HQ703288
Marcia hiantina|HQ703292
Marcia hiantina|HQ703291
Marcia hiantina|HQ703290
Marcia japonica|EF367171
Marcia japonica|HQ703285
Marcia japonica|HQ703286
Marcia japonica|HQ703282
Marcia japonica|HQ703283
Marcia japonica|HQ703284
Paphia dura|DQ184826
Paphia dura|DQ458500
Tapes dorsatus|EF367172
Tapes dorsatus|HQ703229
Tapes dorsatus|HQ703230
Tapes dorsatus|HQ703224
Tapes dorsatus|HQ703228
Tapes dorsatus|HQ703225
Tapes dorsatus|HQ703226
Tapes dorsatus|HQ703227
Venerupis decussatus|DQ184830
Venerupis decussatus|DQ458492
Paphia gallus B|HQ703233
Paphia exarata|EF367170
Paphia gallus B|HQ703235
Paphia gallus B|HQ703234
Paphia gallus A|HQ703232
Paphia sinuosa|HQ703231
Paphia undulata|HQ703246
Paphia undulata|HQ703248
Paphia undulata|HQ703245
Paphia undulata|HQ703244
Paphia undulata|HQ703247
Paphia textile|HQ703236
Paphia textile|HQ703237
Paphia papilionacea|HQ703241
Paphia papilionacea|HQ703243
Paphia papilionacea|HQ703242
Paphia papilionacea|HQ703240
Paphia papilionacea|HQ703239
Paphia papilionacea|HQ703238
Paphia vernicosa|DQ184828
Paphia euglypta|DQ184827
Paphia semirugata|HQ703264
Paphia semirugata|HQ703263
Paphia semirugata|HQ703265
Paphia amabilis|HQ703259
Paphia amabilis|HQ703258
Paphia amabilis|HQ703254
Paphia amabilis|HQ703252
Paphia amabilis|HQ703256
Paphia amabilis|HQ703250
Paphia amabilis|HQ703255
Paphia amabilis|HQ703257
Paphia amabilis|HQ703260
Paphia amabilis|HQ703249
Paphia amabilis|HQ703262
Paphia amabilis|HQ703261
Paphia amabilis|HQ703251
Paphia amabilis|HQ703253

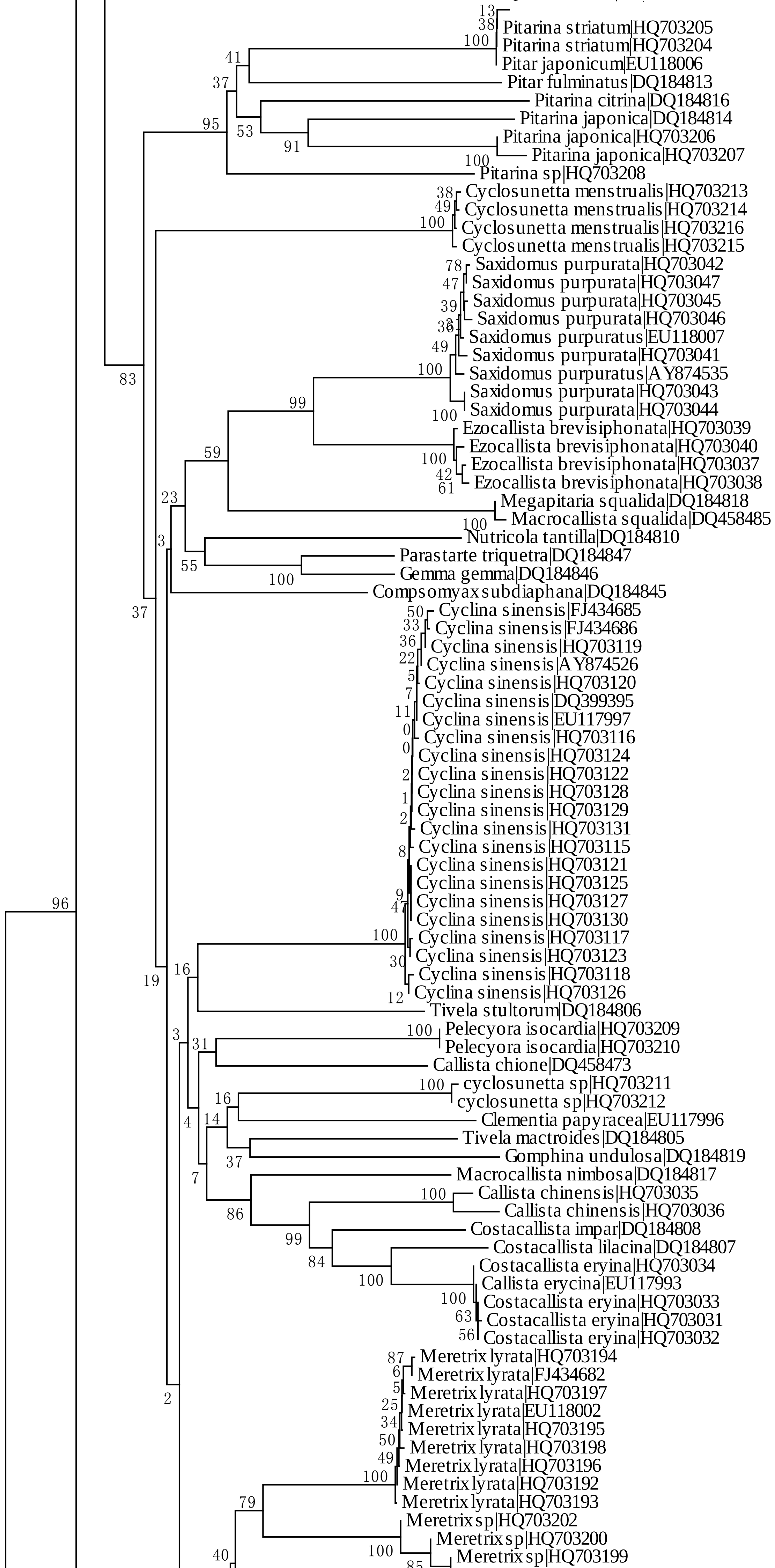

Pitarina striatum|HQ703205
Pitarina striatum|HQ703204
Pitar japonicum|EU118006
Pitar fulminatus|DQ184813
Pitarina citrina|DQ184816
Pitarina japonica|DQ184814
Pitarina japonica|HQ703206
Pitarina japonica|HQ703207
Pitarina sp|HQ703208
Cyclosunetta menstrualis|HQ703213
Cyclosunetta menstrualis|HQ703214
Cyclosunetta menstrualis|HQ703216
Cyclosunetta menstrualis|HQ703215
Saxidomus purpurata|HQ703042
Saxidomus purpurata|HQ703047
Saxidomus purpurata|HQ703045
Saxidomus purpurata|HQ703046
Saxidomus purpuratus|EU118007
Saxidomus purpurata|HQ703041
Saxidomus purpuratus|AY874535
Saxidomus purpurata|HQ703043
Saxidomus purpurata|HQ703044
Ezocallista brevisiphonata|HQ703039
Ezocallista brevisiphonata|HQ703040
Ezocallista brevisiphonata|HQ703037
Ezocallista brevisiphonata|HQ703038
Megapitaria squalida|DQ184818
Macrocallista squalida|DQ458485
Nutricola tantilla|DQ184810
Parastarte triquetra|DQ184847
Gemma gemma|DQ184846
Compsomyax subdiaphana|DQ184845
Cyclina sinensis|FJ434685
Cyclina sinensis|FJ434686
Cyclina sinensis|HQ703119
Cyclina sinensis|AY874526
Cyclina sinensis|HQ703120
Cyclina sinensis|DQ399395
Cyclina sinensis|EU117997
Cyclina sinensis|HQ703116
Cyclina sinensis|HQ703124
Cyclina sinensis|HQ703122
Cyclina sinensis|HQ703128
Cyclina sinensis|HQ703129
Cyclina sinensis|HQ703131
Cyclina sinensis|HQ703115
Cyclina sinensis|HQ703121
Cyclina sinensis|HQ703125
Cyclina sinensis|HQ703127
Cyclina sinensis|HQ703130
Cyclina sinensis|HQ703117
Cyclina sinensis|HQ703123
Cyclina sinensis|HQ703118
Cyclina sinensis|HQ703126
Tivela stultorum|DQ184806
Pelecyora isocardia|HQ703209
Pelecyora isocardia|HQ703210
Callista chione|DQ458473
cyclosunetta sp|HQ703211
cyclosunetta sp|HQ703212
Clementia papyracea|EU117996
Tivela mactroides|DQ184805
Gomphina undulosa|DQ184819
Macrocallista nimbosa|DQ184817
Callista chinensis|HQ703035
Callista chinensis|HQ703036
Costacallista impar|DQ184808
Costacallista lilacina|DQ184807
Costacallista eryina|HQ703034
Callista erycina|EU117993
Costacallista eryina|HQ703033
Costacallista eryina|HQ703031
Costacallista eryina|HQ703032
Meretrix lyrata|HQ703194
Meretrix lyrata|FJ434682
Meretrix lyrata|HQ703197
Meretrix lyrata|EU118002
Meretrix lyrata|HQ703195
Meretrix lyrata|HQ703198
Meretrix lyrata|HQ703196
Meretrix lyrata|HQ703192
Meretrix lyrata|HQ703193
Meretrix sp|HQ703202
Meretrix sp|HQ703200
Meretrix sp|HQ703199

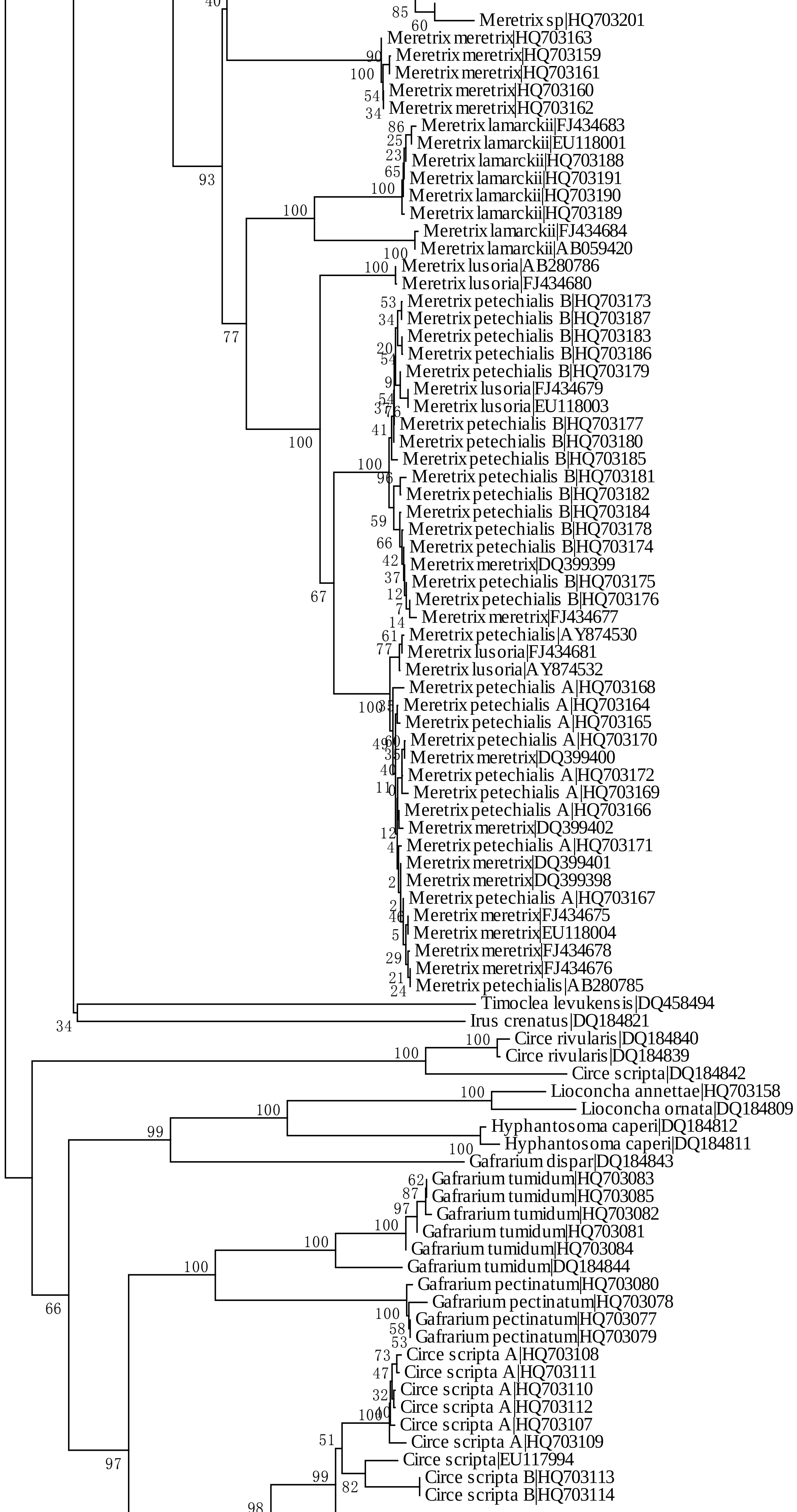

Meretrix sp|HQ703201
Meretrix meretrix|HQ703163
Meretrix meretrix|HQ703159
Meretrix meretrix|HQ703161
Meretrix meretrix|HQ703160
Meretrix meretrix|HQ703162
Meretrix lamarckii|FJ434683
Meretrix lamarckii|EU118001
Meretrix lamarckii|HQ703188
Meretrix lamarckii|HQ703191
Meretrix lamarckii|HQ703190
Meretrix lamarckii|HQ703189
Meretrix lamarckii|FJ434684
Meretrix lamarckii|AB059420
Meretrix lusoria|AB280786
Meretrix lusoria|FJ434680
Meretrix petechialis B|HQ703173
Meretrix petechialis B|HQ703187
Meretrix petechialis B|HQ703183
Meretrix petechialis B|HQ703186
Meretrix petechialis B|HQ703179
Meretrix lusoria|FJ434679
Meretrix lusoria|EU118003
Meretrix petechialis B|HQ703177
Meretrix petechialis B|HQ703180
Meretrix petechialis B|HQ703185
Meretrix petechialis B|HQ703181
Meretrix petechialis B|HQ703182
Meretrix petechialis B|HQ703184
Meretrix petechialis B|HQ703178
Meretrix petechialis B|HQ703174
Meretrix meretrix|DQ399399
Meretrix petechialis B|HQ703175
Meretrix petechialis B|HQ703176
Meretrix meretrix|FJ434677
Meretrix petechialis|AY874530
Meretrix lusoria|FJ434681
Meretrix lusoria|AY874532
Meretrix petechialis A|HQ703168
Meretrix petechialis A|HQ703164
Meretrix petechialis A|HQ703165
Meretrix petechialis A|HQ703170
Meretrix meretrix|DQ399400
Meretrix petechialis A|HQ703172
Meretrix petechialis A|HQ703169
Meretrix petechialis A|HQ703166
Meretrix meretrix|DQ399402
Meretrix petechialis A|HQ703171
Meretrix meretrix|DQ399401
Meretrix meretrix|DQ399398
Meretrix petechialis A|HQ703167
Meretrix meretrix|FJ434675
Meretrix meretrix|EU118004
Meretrix meretrix|FJ434678
Meretrix meretrix|FJ434676
Meretrix petechialis|AB280785
Timoclea levukensis|DQ458494
Irus crenatus|DQ184821
Circe rivularis|DQ184840
Circe rivularis|DQ184839
Circe scripta|DQ184842
Lioconcha annettae|HQ703158
Lioconcha ornata|DQ184809
Hyphantosoma caperi|DQ184812
Hyphantosoma caperi|DQ184811
Gafrarium dispar|DQ184843
Gafrarium tumidum|HQ703083
Gafrarium tumidum|HQ703085
Gafrarium tumidum|HQ703082
Gafrarium tumidum|HQ703081
Gafrarium tumidum|HQ703084
Gafrarium tumidum|DQ184844
Gafrarium pectinatum|HQ703080
Gafrarium pectinatum|HQ703078
Gafrarium pectinatum|HQ703077
Gafrarium pectinatum|HQ703079
Circe scripta A|HQ703108
Circe scripta A|HQ703111
Circe scripta A|HQ703110
Circe scripta A|HQ703112
Circe scripta A|HQ703107
Circe scripta A|HQ703109
Circe scripta|EU117994
Circe scripta B|HQ703113
Circe scripta B|HQ703114

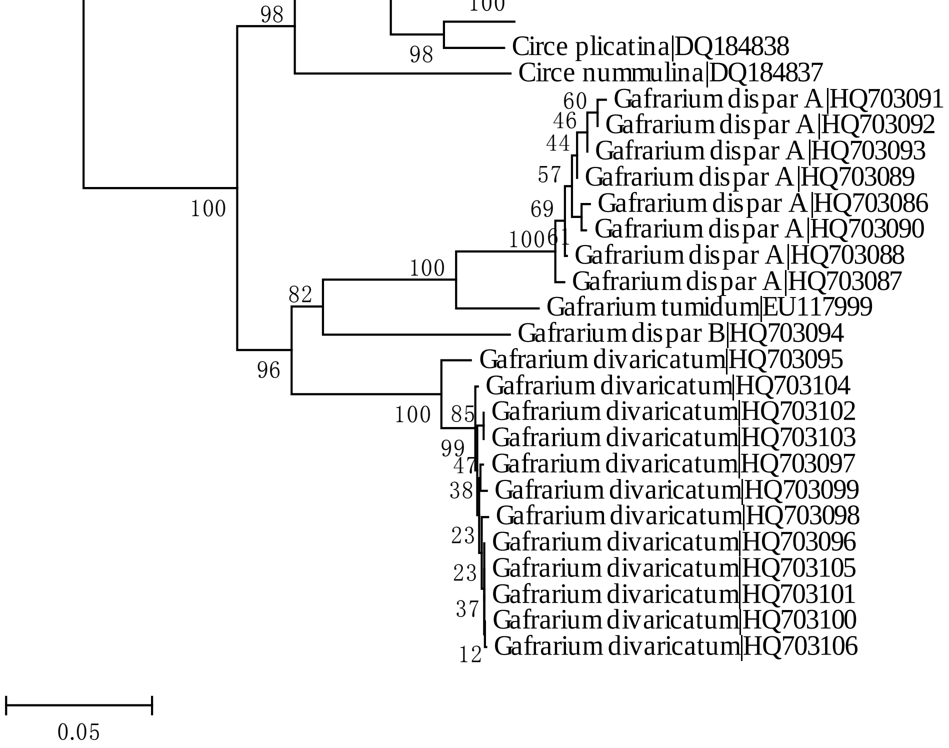

100
98
98
Circe plicatina|DQ184838
Circe nummulina|DQ184837
60
Gafrarium dispar A|HQ703091
46
Gafrarium dispar A|HQ703092
44
Gafrarium dispar A|HQ703093
57
Gafrarium dispar A|HQ703089
69
Gafrarium dispar A|HQ703086
Gafrarium dispar A|HQ703090
100
10061
Gafrarium dispar A|HQ703088
Gafrarium dispar A|HQ703087
100
Gafrarium tumidum|EU117999
82
Gafrarium dispar B|HQ703094
96
Gafrarium divaricatum|HQ703095
Gafrarium divaricatum|HQ703104
100
85
Gafrarium divaricatum|HQ703102
Gafrarium divaricatum|HQ703103
99
47
Gafrarium divaricatum|HQ703097
38
Gafrarium divaricatum|HQ703099
Gafrarium divaricatum|HQ703098
23
Gafrarium divaricatum|HQ703096
23
Gafrarium divaricatum|HQ703105
Gafrarium divaricatum|HQ703101
37
Gafrarium divaricatum|HQ703100
12
Gafrarium divaricatum|HQ703106
0.05
